# Proteomic analysis of stress‐related proteins and metabolic pathways in *Picea asperata* somatic embryos during partial desiccation

**DOI:** 10.1111/pbi.12588

**Published:** 2016-07-14

**Authors:** Danlong Jing, Jianwei Zhang, Yan Xia, Lisheng Kong, Fangqun OuYang, Shougong Zhang, Hanguo Zhang, Junhui Wang

**Affiliations:** ^1^ State Key Laboratory of Tree Genetics and Breeding Key Laboratory of Tree Breeding and Cultivation of State Forestry Administration Research Institute of Forestry Chinese Academy of Forestry Beijing China; ^2^ Department of Biology Centre for Forest Biology University of Victoria Victoria BC Canada; ^3^ State Key Laboratory of Tree Genetics and Breeding Northeast Forestry University Harbin China

**Keywords:** *Picea asperata*, somatic embryo, partial desiccation treatment, proteomics, stress‐related protein

## Abstract

Partial desiccation treatment (PDT) stimulates germination and enhances the conversion of conifer somatic embryos. To better understand the mechanisms underlying the responses of somatic embryos to PDT, we used proteomic and physiological analyses to investigate these responses during PDT in *Picea asperata*. Comparative proteomic analysis revealed that, during PDT, stress‐related proteins were mainly involved in osmosis, endogenous hormones, antioxidative proteins, molecular chaperones and defence‐related proteins. Compared with those in cotyledonary embryos before PDT, these stress‐related proteins remained at high levels on days 7 (D7) and 14 (D14) of PDT. The proteins that differentially accumulated in the somatic embryos on D7 were mapped to stress and/or stimuli. They may also be involved in the glyoxylate cycle and the chitin metabolic process. The most significant difference in the differentially accumulated proteins occurred in the metabolic pathways of photosynthesis on D14. Furthermore, in accordance with the changes in stress‐related proteins, analyses of changes in water content, abscisic acid, indoleacetic acid and H_2_O_2_ levels in the embryos indicated that PDT is involved in water‐deficit tolerance and affects endogenous hormones. Our results provide insight into the mechanisms responsible for the transition from morphologically mature to physiologically mature somatic embryos during the PDT process in *P. asperata*.

## Introduction

The complete process of the vegetative propagation technique, somatic embryogenesis, in conifer includes embryonic callus initiation, proliferation, somatic embryo maturation and germination (Stasolla and Yeung, 2003). Among these phases, germination/conversion is regarded as the most important step to obtain plantlets; this determines the success of this technique. Morphologically, mature conifer somatic embryos cannot germinate or convert into viable plantlets unless the embryos undergo partial desiccation treatment (PDT) (Stasolla *et al*., 2002). This treatment has been used effectively to improve the germination/conversion of somatic embryos in *Picea abies* (Bozhkov and Von Arnold, 1998; Find, 1997; Högberg *et al*., 1998), *P. rubens* (Harry and Thorpe, 1991), *P. glauca* (Attree *et al*., 1991; Kong and Yeung, 1995), *P. mariana* (Beardmore and Charest, 1995) and the *Pinus* species, *P. patula* (Jones and van Staden, 2001), *P. thunbergii*,* P. densiflora* and *P. armandii* var. *amamiana* (Maruyama and Hosoi, 2012), as well as *Abies nordmanniana* (Nørgaard, 1997; Salajova and Salaj, 2001; Vooková and Kormuťák, 2006).

Partial desiccation treatment that caused a gradual and limited loss of moisture content in conifer somatic embryos was first reported by Roberts (Roberts *et al*., 1990). Some physiological and metabolic changes during PDT have been reported in conifer somatic embryos (Dronne *et al*., 1997; Find, 1997; Kong and Yeung, 1995; Stasolla *et al*., 2001). The somatic embryos of *P. mariana* and *P. glauca* were dried at 97% or 88% relative humidity in the dark to reach a water content of 0.23 g H_2_O/g d.wt before high rates of embryo germination/conversion were achieved (Bomal and Tremblay, 2000). The increased germination by PDT has been attributed to a substantial decrease in the endogenous levels of abscisic acid (ABA) (Find, 1997; Liao and Juan, 2015). Somatic embryos of white spruce produce less ethylene during the drying process (Kong and Yeung, 1994), while purine and pyrimidine metabolism is enhanced during PDT (Stasolla *et al*., 2001). The fact that conifer somatic embryos can germinate and convert into viable plantlets only after PDT indicates that changes in gene expression may occur in these embryos during the process of PDT, resulting in the synthesis of sufficient stress‐related proteins and germination‐associated proteins. At present, the regulatory mechanisms underlying PDT remain unclear and this hypothesis needs further research.

Proteomics, which provides a global analysis of protein fluctuations, is a more effective technique than transcriptomics for inferring the role of stress‐related proteins (Sano *et al*., 2013). The isobaric tags for relative and absolute quantitation (iTRAQ) system is currently one of the most robust mass spectrometry techniques. This technology compares proteins on the basis of iTRAQ‐tagged peptides, allowing identification and accurate quantification of proteins from multiple samples within dynamic ranges of protein abundance (Casado‐Vela *et al*., 2010; Chu *et al*., 2015; Nogueira *et al*., 2012). Comparative analysis of proteomics can provide further insight into the mechanisms regulating important processes during conifer somatic embryogenesis. The proteome analysis of Lippert *et al*. (2005) revealed that differentially accumulated proteins are involved in a variety of cellular processes in early somatic embryogenesis in *P. glauca*. During the somatic embryo development of *Larix principis‐ruprechtii*, functional analysis of proteomics showed that the differentially accumulated proteins involved in primary metabolism, phosphorylation and oxidation reduction are up‐regulated (Zhao *et al*., 2015). Although changes in the transcript levels of many genes have been reported using DNA microarrays during the maturation phase of somatic embryos in white spruce (Stasolla *et al*., 2003), global protein fluctuations in conifer somatic embryos during PDT have not yet been investigated. To identify stress‐related proteins, germination‐associated proteins, and metabolic pathways in conifer somatic embryos during PDT, proteomic analysis combined with measures of the physiological changes in the embryos is thus required.

*Picea asperata* Mast is a widely distributed native spruce in China. It has attracted increasing attention with regard to afforestation in barren regions due to its outstanding wood properties and adaptability (Fu *et al*., 1999; Luo *et al*., 2006). Our well‐established somatic embryogenesis system in *P. asperata* provides plant materials of identical genotypes and highly synchronized embryos for accurate proteomics comparison during PDT. In this study, using embryogenic cell line 1931, we investigated the differences and changes in *P. asperata* somatic embryos at various stages (i.e. cotyledonary embryos before, during and after PDT) using iTRAQ‐based proteomic and physiological analyses. With this most extensive proteomics analysis for *P. asperata* somatic embryos, we reveal important stress‐related proteins and metabolic pathways that are associated with PDT in conifer somatic embryos.

## Results

### Effects of PDT on the morphology of *P. asperata* somatic embryos and their germination

**Figure 1:**
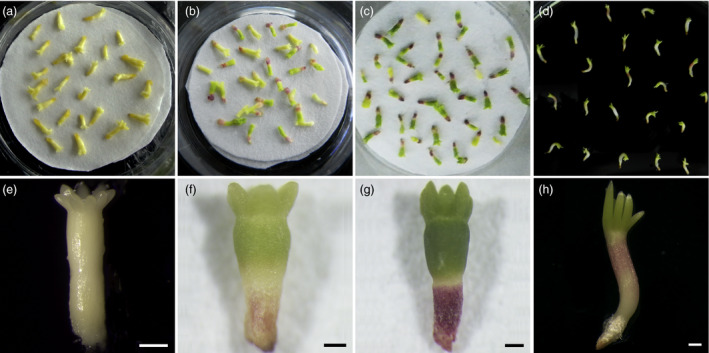
Effects of partial desiccation treatment (PDT) on the morphology of *P. asperata* somatic embryos. (a) and (e) Embryos before PDT were light yellowish on day 0 (D0) without colour change. (b) and (f) Embryos under PDT for 7 days (D7) had green cotyledons, green hypocotyls and red radicles. (c) and (g) Embryos under PDT for 14 days (D14) had dark green cotyledons, dark green hypocotyls and red radicles. (d) and (h) Embryos in germination medium on day 1 after PDT for 7 days (G1) showed growth of cotyledons and elongation of radicles and hypocotyls. All scale bar, 500 μm.

To determine the effects of PDT on these embryos, the morphological characteristics of developing the embryos were analysed on days 0 (D0), 7 (D7), 14 (D14) of PDT and on germination day 1 after 7 days of PDT (G1). The mature somatic embryos were yellowish after separation from differential medium. The cotyledons were completely open and arranged circularly on the shoot apical pole (Figure 1a, e). During PDT, the embryos shrunk rapidly on the first day, and then the hypocotyls constantly thickened and elongated. On D7, the radicles became red, while the cotyledons and hypocotyls turned green (Figure 1b, f). After desiccation for 14 days, the cotyledons were dark green and closely attached to each other, while the hypocotyls elongated significantly and the radicles became dark red (Figure 1c, g). On G1, the hypocotyls elongated significantly and their sizes increased longitudinally. The radicles elongated markedly and accompanied by a colour change to white (Figure 1d, h).

**Figure 2:**
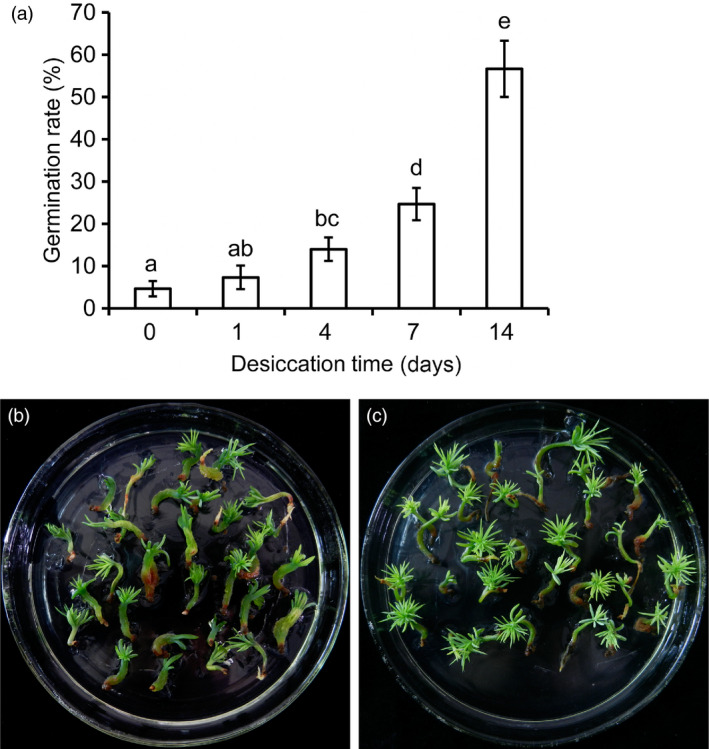
Effects of partial desiccation treatment (PDT) on the germination of *P. asperata* somatic embryos. (a) The germination rates of embryos after PDT for different times. Mean ± SD,* n* = 5. Significant differences are indicated by different letters (*P* < 0.05). (b) Photograph of embryos germinating without PDT. Most of the germinants were abnormal with clear hyperhydricity and poor radicle development. (c) Photograph of embryos germinating after 14 days of PDT. The germinants were strong with well‐developed roots and shoots.

A germination standard (Liao and Juan, 2015) was used to assess the germination performance after the embryos were placed on germination medium. From D0 to D14, the germination rate increased significantly corresponding to the duration of partial desiccation (Figure 2a). Compared with embryos without PDT, the germination rate increased significantly from 4.67% to 56.83% after 14 days of PDT. Without PDT, most of the hypocotyls became hyperhydric, and the radicles turned dark brown during germination (Figure 2b). After 7 days of PDT, embryo germination was stimulated with little hyperhydricity of the germinants and high germination rates. The plantlets converted from embryos after PDT showed hypocotyl extension and needle development at the shoot apical end (Figure 2c).

### Identification of stress‐related proteins and metabolic pathways in *P. asperata* somatic embryos

To determine the protein fluctuations during PDT, the total proteins in embryos on D0, D7, D14 and G1 were extracted and their profiles were explored using the iTRAQ technique. A total of 347 380 spectra were generated; 34 301 (9.87%) matched known peptides according to Mascot software; and 28 651 (83.53%) matched unique peptides. Ultimately, 10 216 peptides, 9297 (91.00%) unique peptides and 2773 proteins were identified. Meanwhile, the distributions of the lengths and numbers of peptides, mass and sequence coverage of the proteins, and the repeatability of replicates were assessed (Figures S1 and S2).

The annotated proteins were classified into three groups (cellular component, molecular function and biological process) on the basis of Gene Ontology (GO) enrichment analysis. The main cellular components were classified into cell (23.31%), cell part (23.31%), organelle (19.18%) and others (Figure S3). The molecular functions of proteins were mainly focused on catalytic activity (43.89%) and binding (41.25%) (Figure S4). The biological processes were mainly metabolic process (19.97%), cellular process (19.14%), response to stimulus (10.21%) and others (Figure S5).

**Figure 3:**
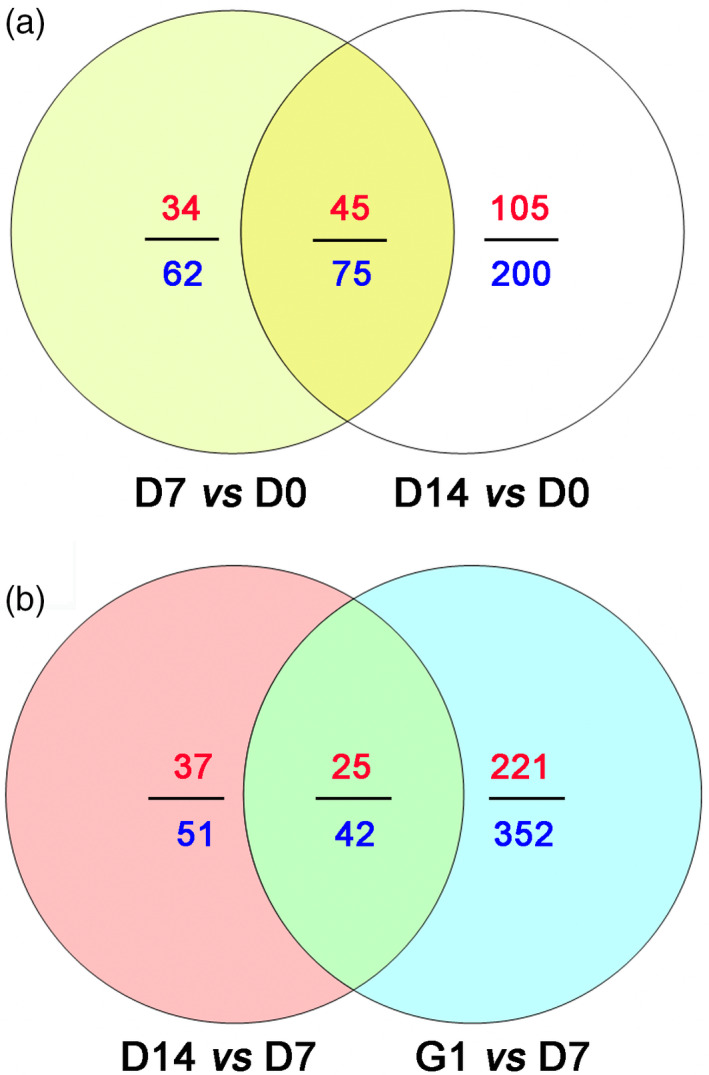
Venn diagram of differentially accumulated proteins in D7 *vs* D0 and D14 *vs* D0, D14 *vs* D7 and G1 *vs* D7. (a) Differentially accumulated proteins in D7 *vs* D0 and D14 *vs* D0. (b) Differentially accumulated proteins in D14 *vs* D7 and G1 *vs* D7. The numbers of differentially accumulated proteins are represented in blue. The numbers of up‐regulated differentially accumulated proteins are indicated in red.

Proteomic analysis of D7 *vs* D0, D14 *vs* D0, D14 *vs* D7 and G1 *vs* D7 was used to detect stress‐related proteins in the embryos under PDT. A total of 636 proteins showed significant difference based on a 1.5‐fold change at *P* < 0.05 (Figure S6). Comparisons of D7 *vs* D0 and D14 *vs* D0 showed a total of 337 differentially accumulated proteins, of which 66 (19.58%) were associated with stress tolerance (Figure 3a and Table S1). These stress‐related proteins were mainly involved in osmosis, endogenous hormones, antioxidative proteins, molecular chaperones, defence‐related proteins, pyrimidine metabolism and embryogenesis‐specific protein.

According to the physiological functions of stress‐related differentially accumulated proteins, auxin‐repressed 12.5 kDa protein, allene oxide synthase and abscisic stress‐ripening protein were classified as endogenous hormones in the embryos under PDT. Three types of aquaporins (TIP2‐1, TIP1‐1 and PIP type) were associated with water transport by osmosis to prevent desiccation of the embryos during PDT. We identified 5 classes of defence‐related proteins: pathogenesis‐related protein, wound‐induced protein, basic endochitinase, chitinase and osmotin. During PDT, 35 differentially accumulated proteins were enriched in antioxidative proteins, associated with the response to reactive oxygen species to diminish cytotoxic damage such as DNA damage, protein modification, lipid peroxidation and de‐esterification (Table S1). Meanwhile, phosphoenolpyruvate carboxykinase, aldose 1‐epimerase, NAD‐dependent malic enzyme 62 kDa isoform, sucrose synthase 2 and xyloglucan endotransglucosylase/hydrolase protein A were identified as being involved in carbohydrate metabolism. The small heat‐shock protein, class I heat‐shock protein, splicing factor, heat‐shock 70 kDa protein 10 and 18.1 kDa class I heat‐shock protein were found to be molecular chaperones and involved in desiccation tolerance of the embryos. In addition, pyrimidine metabolism‐related protein (deoxyuridine 5′‐triphosphate nucleotidohydrolase) and embryogenesis‐specific protein (provicilin, stem‐specific protein, tubulin and 12S seed storage protein) were also identified.

Comparative proteomics of D14 *vs* D7 and G1 *vs* D7 were further analysed to evaluate the relevance of germination after PDT (G1) and a longer period of PDT (D14). From the sum of the numbers of differentially accumulated protein in both cases, we detected 445 such proteins, of which 52 (11.69%) were involved in photosynthesis (Figure 3b and Table S2). Among these photosynthesis‐related proteins, 39 were identified in the D14 *vs* D7 comparison, while 28 were identified in the G1 *vs* D7 comparison. Meanwhile, the 15 photosynthesis‐related proteins that were enriched in both D14 *vs* D7 and G1 *vs* D7 were mainly associated with light harvesting, chlorophyll synthesis and photosynthetic protection (Table S2).

**Figure 4:**
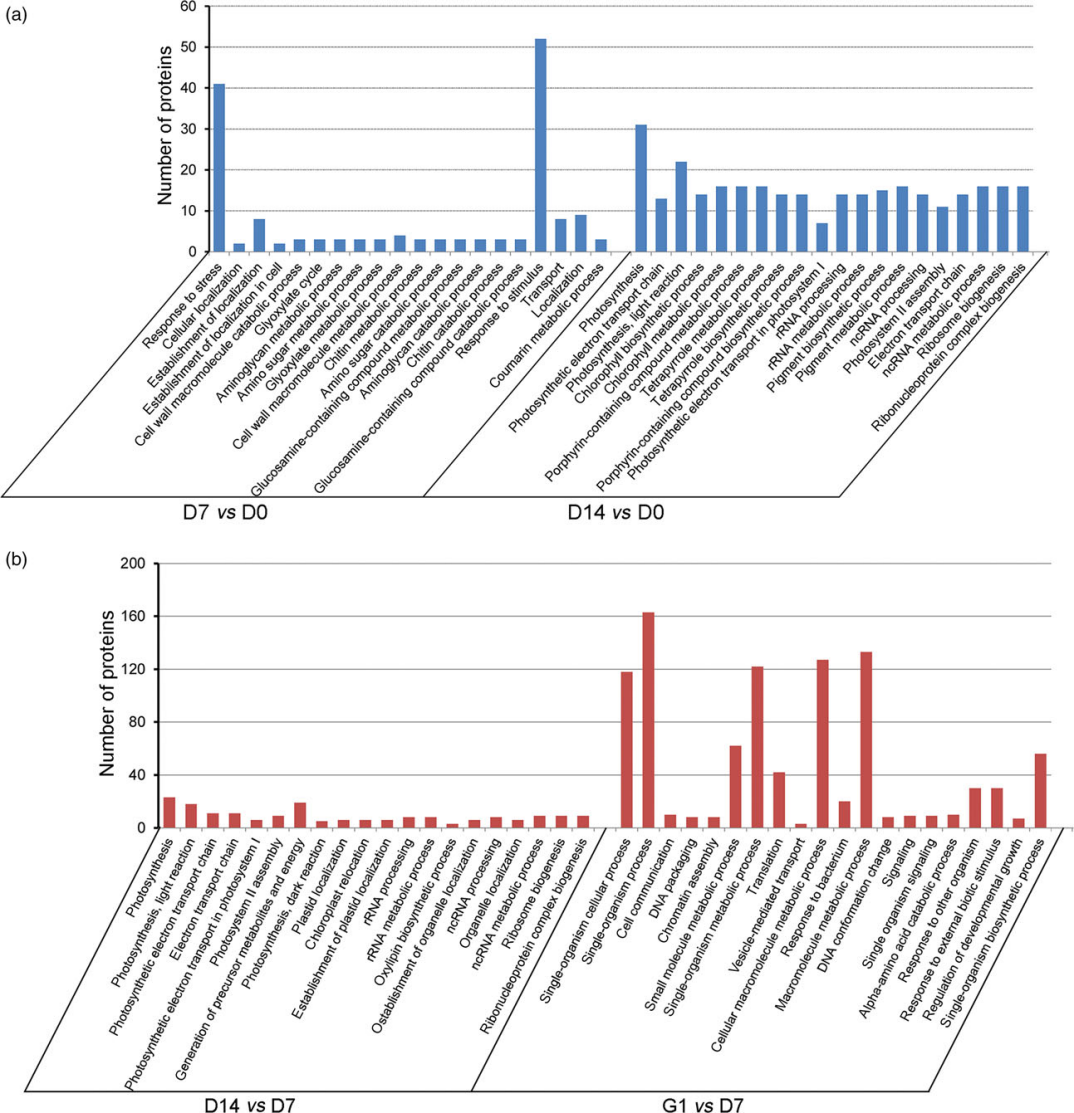
Gene Ontology (GO) enrichment analysis of differentially accumulated proteins. (a) Biological process classification for D7 *vs* D0 and D14 *vs* D0. (b) Biological process categories for D14 *vs* D7 and G1 *vs* D7.

To better visualize the differences in the metabolic pathways of the embryos during PDT, these differentially accumulated proteins were classified on the basis of GO enrichment analysis (Figure 4). The main biological functional categories for D7 *vs* D0 were response to stress, glyoxylate cycle, chitin metabolic process and response to stimulus. The biological processes for D14 *vs* D0 were mainly classified into photosynthesis categories, including photosynthetic electron transport chain, light reaction, chlorophyll biosynthetic process, tetrapyrrole biosynthetic process, porphyrin‐containing compound biosynthetic process and photosynthetic electron transport in photosystem I (Figure 4a). The main biological functional categories for D14 *vs* D7 were photosynthesis, including light reaction, photosynthetic electron transport chain, electron transport chain, photosynthetic electron transport in photosystem I and photosystem II assembly. The biological processes for G1 *vs* D7 were mainly single‐organism process, cell communication, DNA packaging, chromatin assembly, small‐molecule metabolic process and translation (Figure 4b).

### Changes in stress‐related proteins in *P. asperata* somatic embryos during PDT

**Figure 5:**
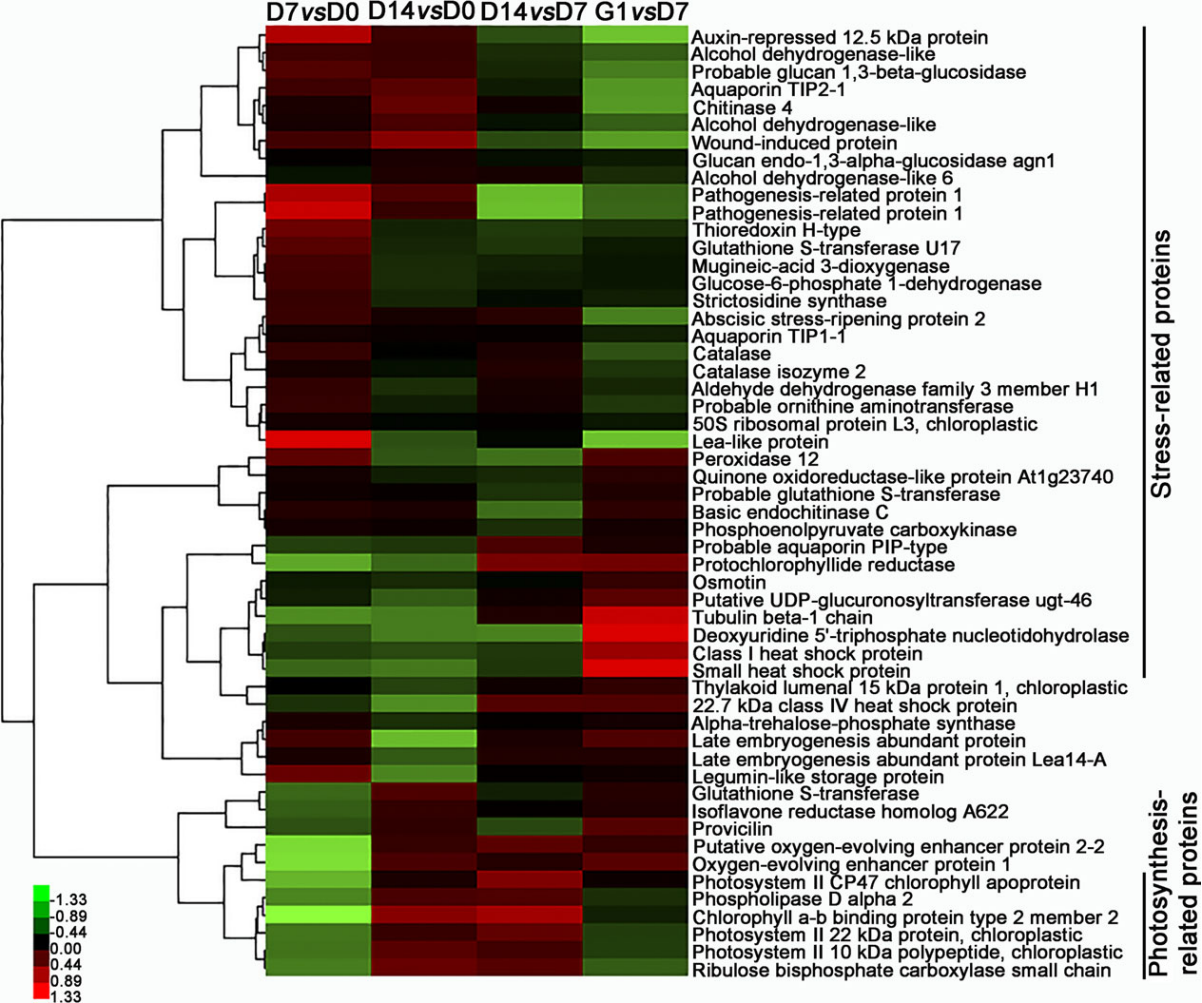
Heat map of normalized fold changes in accumulated proteins in D7 *vs* D0, D14 *vs* D0, D14 *vs* D7 and G1 *vs* D7.

Fold changes in the protein profile of embryos during PDT were further analysed to estimate the stress‐related proteins associated with water‐deficit tolerance. The fold changes of stress‐related proteins accumulated in both D7 *vs* D0 and D14 *vs* D0 are shown in Table S3. Among these accumulated proteins, auxin‐repressed 12.5 kDa protein and abscisic stress‐ripening protein 2 were significantly up‐regulated on D7 and D14. Compared with D0, aquaporin TIP2‐1 was increased by 1.75‐fold on D7 and 3.56‐fold on D14. Four defence‐related proteins, including wound‐induced protein, osmotin, chitinase 4 and basic endochitinase C, were up‐regulated 4.10‐, 1.53‐, 2.60‐ and 2.32‐fold on D7, and 8.20‐, 2.10‐, 5.30‐ and 3.29‐fold on D14 relative to D0, respectively. Twenty‐eight accumulated proteins associated with antioxidation were significantly increased to reduce the effects of deleterious reactive oxygen species during PDT. For example, catalase and catalase isozyme 2 were increased 1.61‐ and 1.56‐fold on D7, and 1.92‐ and 2.06‐fold on D14, respectively. The change in catalase isozyme protein corresponded well with the catalase activity assays (Figure S7). Furthermore, we analysed changes in differentially accumulated proteins for D7 *vs* D0, D14 *vs* D0, D14 *vs* D7 and G1 *vs* D7 (Figure 5). Compared with D0, the stress‐related proteins were up‐regulated on D7 and D14, while the photosynthesis‐related proteins were significantly up‐regulated on D14.

### Photosynthesis metabolism proteins and protein–protein interaction networks of differentially accumulated proteins

A total of 47 differentially accumulated proteins were significantly enriched in the photosynthesis pathway and were directly associated with photosynthesis (Table S2). Among these proteins, 36 were significantly up‐regulated from D7 to D14, while 29 were up‐regulated from D7 to G1. Notably, photosystem II, photosynthetic electron transport and F‐type ATPase proteins were involved in photosynthetic metabolism under PDT (Figure S8). These patterns of differentially accumulated proteins showed that the embryos gradually carried out photosynthesis from D7.

A total of 1047 proteins were identified using *Arabidopsis* interaction data to evaluate the protein–protein interaction networks of the *P. asperata* somatic embryos under PDT. The protein interactions corresponded well to those from *Arabidopsis*. The 562 pairs of protein–protein interactions were used to build 498 vertices in the network (Figures S9–S12). Collectively, 13 interacting proteins were significantly differentially accumulated in the different PDT groups (Table 1). The interacting proteins that were up‐regulated during PDT and the germination process were mainly associated with photosynthesis, glyoxylate and dicarboxylate metabolism, xenobiotics metabolism, protein processing in the endoplasmic reticulum and carbon fixation in photosynthetic organisms. However, proteins of the ribosome pathway were up‐regulated during the PDT process, but down‐regulated during the germination process, while proteins of the proteasome and protein‐processing pathways were down‐regulated during the PDT process, but up‐regulated during the germination process.

**Table 1:** Common expression of protein–protein interaction among the different treatment

Accession ID	KEGG_Pathway	D7 *vs* D0	D14 *vs* D0	D14 *vs* D7	G1 *vs* D7
Pasi_224284112	Photosynthesis	Up	Up	Up	Up
Pasi_224285151	Photosynthesis	Up	Up	Up	
Pasi_116780862	Glyoxylate and dicarboxylate metabolism	Up	Up	Up	Up
Pasi_224286155	Proteasome	Down		Down	Up
Pasi_116793156	Protein processing in endoplasmic reticulum	Down		Down	Up
Pasi_224286155	Proteasome	Down		Down	Up
Pasi_116792087	Metabolism of xenobiotics by cytochrome P450	Up		Up	Up
Pasi_224286188	Protein processing in endoplasmic reticulum		Up	Up	Up
Pasi_148907259	Protein processing in endoplasmic reticulum		Up	Up	Up
Pasi_116786455	Carbon fixation in photosynthetic organisms			Up	Up
Pasi_116781903	RNA transport			Down	Up
Pasi_224285264	Ribosome	Up			Down
Pasi_294464103	RNA polymerase	Down		Down	

### Effects of PDT on water, ABA, indoleacetic acid (IAA) and H_2_O_2_ in *P. asperata* somatic embryos

**Figure 6:**
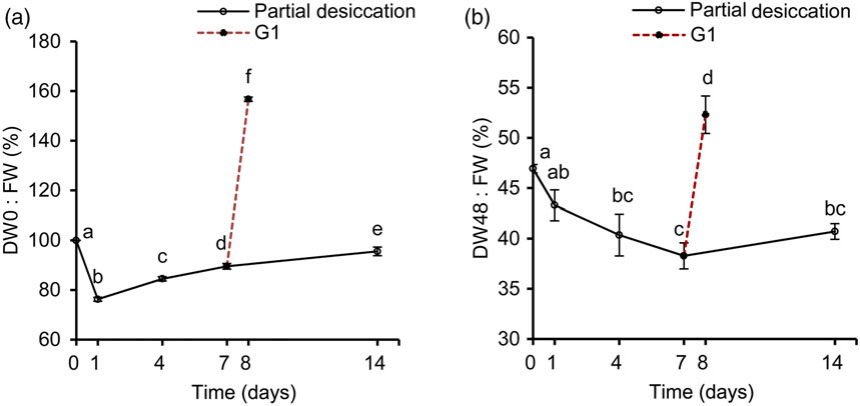
Changes of the water content in *P. asperata* somatic embryos during partial desiccation treatment (PDT). (a) Effect of the desiccation period on fresh weight. FW, fresh weight before PDT; DW0, fresh weight during PDT. (b) Effect of the desiccation period on dry weight. DW48, dry weight during PDT. Mean ± SD,* n* = 4. Significant differences are indicated by different letters (*P* < 0.05).

To obtain an accurate understanding of the stress‐related proteins and physiological changes in these embryos during PDT, we analysed their water, ABA, IAA and H_2_O_2_ content at D0, D1, D4, D7, D14 and G1. The water content declined (*P* < 0.05) drastically on the first day and then increased gradually (Figure 6a). On D1, the embryos were relatively dry as a result of rapid water loss to only 76.28% of the original fresh weight. However, the fresh weight recovered to 95.58% on D14. The embryos absorbed water rapidly from the culture medium when the embryos, after 7 days of PDT, were placed on germination medium for 1 day (i.e. G1), resulting in a fresh weight of 1.57‐fold the initial value.

The dry weight of the embryos changed drastically with different durations of desiccation (Figure 6b). From D0 to D7, the dry weight declined due to transfer of the embryos from culture medium to an empty plate with filter paper. After D7, their dry weight began to increase until D14. Dry matter accumulated strongly on G1.

**Figure 7:**
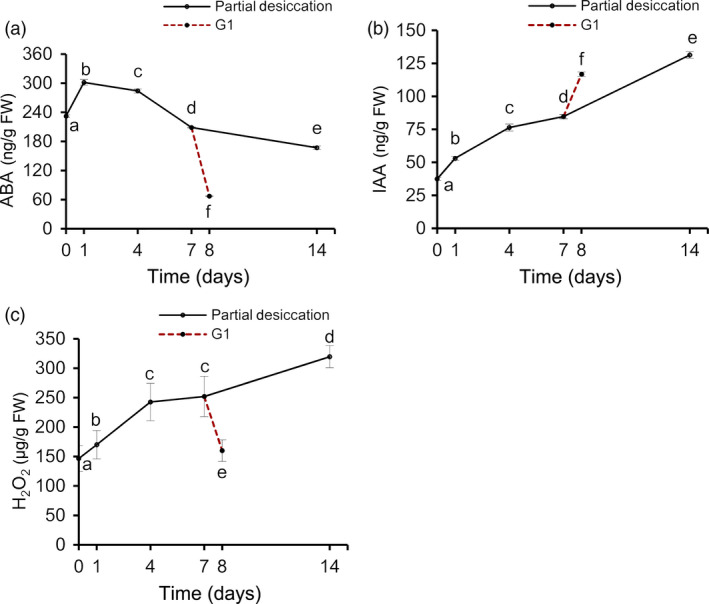
Changes in abscisic acid (ABA), indoleacetic acid (IAA) and H_2_O_2_ contents in *P. asperata* somatic embryos during PDT. (a) ABA content. (b) IAA content. (c) H_2_O_2_ content. Mean ± SD,* n* = 4. Significant differences are indicated by different letters (*P* < 0.05).

During PDT, the concentrations of ABA in the embryos increased slightly on D1, and decreased thereafter (Figure 7a), reaching the lowest level on D14. The IAA level increased during PDT (Figure 7b). The H_2_O_2_ levels increased significantly during PDT, but then decreased quickly on G1 (Figure 7c).

## Discussion

**Figure 8:**
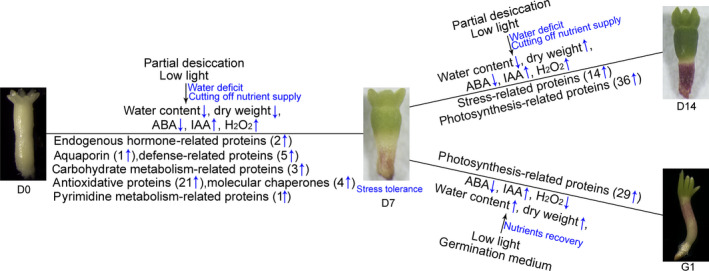
Schematic of changes in the regulatory proteins in response to partial desiccation treatment in *P. asperata* somatic embryos.

In this study, a large number of *P. asperata* somatic embryos of the same genetic background were used to investigate the effects of PDT on the proteome. The morphological, iTRAQ‐based proteomic and physiological analyses highlighted the importance of PDT in stimulating embryo germination. This enabled us to identify the regulatory proteins and to clarify the molecular mechanisms underlying the PDT process. The results suggest that PDT acts by increasing the stress‐related proteins in *P. asperata* somatic embryos that are deficient in water and nutrients. These proteins promote the transformation of these embryos from morphological maturity to physiological maturity and further induce photosynthesis in low light (Figure 8).

### Proteins associated with PDT in *P. asperata* somatic embryos

This work confirms that somatic embryos of *P. asperata*, similar to other conifers (Hazubska‐Przybył *et al*., 2015; Percy *et al*., 2001), suffer from water‐deficit stress under PDT. Comparative proteomics analysis of these embryos during PDT revealed that significantly differentially accumulated proteins are mainly involved in the biological regulation of water, plant hormones and the stress response. Furthermore, some important proteins, such as aquaporins, auxin‐repressed proteins, catalase and heat‐shock proteins, might play important roles during PDT, based on their physiological functions. Here, we found that the aquaporins (TIP2‐1, TIP1‐1 and PIP type) were significantly up‐regulated during PDT; these proteins are important for water content and light induction. Similarly, previous studies have shown that aquaporin forms a ‘tunnel’ in the cell membrane to regulate the water transport under stress conditions (Boursiac *et al*., 2005), and can be induced by light (Loqué *et al*., 2005). As well as lowering the activation energy of water transport, aquaporin also enhances the permeability of the plasma membrane (Leitão *et al*., 2012).

Auxin‐repressed proteins are involved in the response to salicylic acid signalling and are induced by IAA (Shi *et al*., 2013). In our work, auxin‐repressed protein was significantly up‐regulated under PDT but down‐regulated on G1, suggesting that the embryos had entered a stress state that was quickly released in the germination stage. A previous report also indicated that auxin‐repressed protein is induced by abiotic stresses, and involved in growth arrest, possibly by inhibiting cell elongation (Lee *et al*., 2013). As a lytic enzyme of H_2_O_2_, catalase can purge active oxygen to reduce cell damage and its activity is markedly enhanced in the dried seed (Bailly, 2004; Berjak, 2006). In the somatic embryos of *P. asperata* during PDT, catalase remained at high levels on D7 and D14, coinciding with H_2_O_2_ content.

In our study, heat‐shock proteins (Hsps) were up‐regulated during the desiccation period; this might be related to rising H_2_O_2_ levels and embryonic development. However, Hsp22.7 belonged to the Hsp20 family and was down‐regulated under PDT (Figure 5). Taken together, the four kinds of Hsps serving as molecular chaperones were up‐regulated on G1, suggesting that they might help the protein refolding during germination. Previously, Hsps and small‐molecule Hsps, which usually act as molecular chaperones, had been also reported to be involved in the stress response (Sun *et al*., 2002) and the development of seed ripening (Wehmeyer *et al*., 1996), as well as the repair and refolding of damaged proteins, thus protecting cells from damage by the stress and assisting seed or embryo maturation.

### Proteins associated with embryo development and photosynthesis during PDT and germination

During PDT, chitinase was significantly up‐regulated; this might be associated with physiological maturity of the somatic embryos in *P. asperata*. By contrast, in somatic embryos of *P. abies*, chitinase (class IV) has been reported to promote their transformation from embryonic cell mass (Wiweger *et al*., 2003). It was also reported that chitinase was increased at the maturation stage of zygotic embryos in *Araucaria angustifolia* (Dos Santos *et al*., 2006). Notably, glutamine synthase, which is involved in the development of somatic embryos, was up‐regulated during the desiccation and germination in *P. asperata* somatic embryos. As reported for the late seed development of the Brazilian Pine, glutamine synthase accumulated in the early cotyledonary stage; it could facilitate the transformation of glutamate to glutamic acid and be acted as protein markers of embryonic maturity (Balbuena *et al*., 2009).

The level of β‐tubulin, a structural protein, is at its lowest in dry mature seeds. This protein has the highest expression during the dormancy release of Norway maple embryos and can be used as the indicator for dormancy breaking (Pawłowski *et al*., 2004). In *P. asperata* somatic embryos, β‐tubulin was significantly up‐regulated during PDT and germination, indicating that these embryos might be switched to germination stage after 7 days of PDT. The same trend has been reported in the process of seed germination in *Arabidopsis* (Chibani *et al*., 2006).

A major effect of PDT on the *P. asperata* somatic embryos was up‐regulation of photosynthesis‐related proteins. This could explain the increase in dry weight of the embryos after 7 days of PDT, demonstrated that organic matter was accumulating. Meanwhile, the photosynthesis‐related proteins may be up‐regulated to match the requirements of nutrient‐deficit stress, as well as indicating that the embryos have entered the germination stage.

### Physiological responses of *P. asperata* somatic embryos to PDT

Many essential physiological processes for conifer somatic embryo development are affected by stress, exhibiting various defence mechanisms (Robinson *et al*., 2009; dos Santos *et al*., 2016; Zhao *et al*., 2015). In accordance with the changes in stress‐related proteins during the PDT process, the water content in the embryos decreased drastically due to water absorbance by the dry filter paper. Soon afterwards, as the filter paper constantly absorbed moisture from the air, the water content of the *P. asperata* somatic embryos increased. These sharp changes in water content were associated with stress‐related proteins such as aquaporins, suggesting that these embryos suffered water‐deficit stress under PDT. It is known that the cellular messenger H_2_O_2_ causes stress and induces catalase during somatic embryogenesis in *Larix leptolepis* (Zhang *et al*., 2010). The rapidly increased H_2_O_2_ content in *P. asperata* somatic embryos during PDT indicates that these embryos were under oxidative stress and that this was associated with the changes in antioxidative proteins. The stress was released quickly during germination, because the H_2_O_2_ content of the embryos decreased significantly, with just 1 day of germination treatment (G1).

As an important osmotic regulator, ABA content changes when plant embryos are subjected to stress (Xiong and Zhu, 2003). At the beginning of PDT (D1), *P. asperata* somatic embryos suffered a strong desiccation stress, and the ABA content increased significantly. Then, the ABA content tended to decrease after D1, which might be due to rapid adaptation to the PDT condition with increasing humidity. However, our ABA results differed slightly from those of Find (1997), Liao and Juan (2015), probably because we carried out PDT under low light conditions. In somatic embryos of Norway spruce during PDT, the increasing germination frequency has been attributed to a substantial decrease in the ABA content, and one effect of PDT may be the breakdown of endogenous ABA (Find, 1997). On day 7 of PDT, the ABA content of *Picea morrisonicola* somatic embryos is substantially decreased (Liao and Juan, 2015). Dronne *et al*. (1997) also pointed out that PDT decreased the ABA content in hybrid larch somatic embryos. However, Kong (1994) found that PDT does not cause an increase in IAA concentration in white spruce somatic embryos. The increase in IAA concentration in our study may be due to the effect of light on PDT. Higher IAA concentrations could benefit embryo germination in addition to lower ABA concentrations after PDT.

## Experimental procedures

### Plant materials

Highly synchronized somatic embryos of *P. asperata* were obtained from embryogenic cell line 1931, which was initiated from an immature zygotic embryo of an elite mother tree in the National Spruce Germplasm Bank of China, in Gansu province. This cell line had been cultured in liquid medium for 1 year. This medium was consisted of half‐strength salts of Litvay medium (LM) (Litvay *et al*., 1985), 1% sucrose (Beijing, China), 0.1% casein enzymatic hydrolysate (Sigma, St. Louis, MO), 10 μm 2,4‐dichlorophenoxyacetic acid (Sigma) and 5 μm 6‐benzylaminopurine (Sigma), supplemented with 3.42 mm filter‐sterilized L‐glutamine (Sigma) at pH 5.8. Erlenmeyer flasks (250 mL) containing 80 mL liquid medium were used to culture the embryogenic tissue. The flasks of tissue were placed on a gyratory shaker (110 rpm) and cultured in darkness at 24 ± 1 °C. Suspension cultures were transferred to fresh medium every 12 days. Before being cultured on maturation medium for embryonic differentiation, embryogenic tissue was transferred to a piece of sterile filter paper (Whatman, Kent, UK) and the liquid medium was removed using a vacuum pump. Then, the filter paper with embryogenic tissue was transferred onto maturation medium. This medium contained half‐strength salts of LM, 61 μm filter‐sterilized (±) *cis, trans*‐ABA (Gibco‐BRL, Gaithersburg, MD), 5% polyethylene glycol 4000 (PEG4000, Merck, Darmstadt, Germany), 3% sucrose, 0.1% activated charcoal (Sigma) and 0.4% gellan gum (Sigma), supplemented with 3.42 mm filter‐sterilized L‐glutamine, 0.1% casein hydrolysate (Sigma), at pH 5.8. The cultures were kept in the dark at 24 ± 1 °C for 7 weeks. In total, more than 30 000 highly synchronized somatic embryos were obtained for later use.

### Partial desiccation of *P. asperata* somatic embryos

For PDT, each batch of ~30 somatic embryos was transferred onto two layers of dry sterile filter paper (Whatman) in a small plastic Petri dish (35 × 12 mm) without a lid. Three of the dishes containing embryos were placed in a large Petri dish (90 × 15 mm) with 10 mL sterile deionized water. The large Petri dish was covered by a lid and sealed with parafilm, then incubated in a cultivation room at 24 ± 1 °C. Unlike PDT of conifer somatic embryos in the dark, we placed *P. asperata* somatic embryos under a 16‐h photoperiod with a low light intensity of ~15 μmol/m^2^/s (LED fluorescent tubes) for 0 (D0), 1 (D1), 4 (D4), 7 (D7) and 14 days (D14). At the same time, some of the somatic embryos were partially desiccated for 7 days and then germinated at a low light intensity of ~15 μmol/m^2^/s (LED fluorescent tubes) for 1 day (G1).

After various treatments, embryos were collected, immediately frozen in liquid nitrogen and stored at −80 °C for water content, ABA, IAA and H_2_O_2_ analysis. Four independent biological replicates were acquired. Meanwhile, somatic embryos from D0, D7, D14 and G1 were assayed for iTRAQ analysis with two independent biological repeats for each treatment.

### Plantlet regeneration capacity of *P. asperata* somatic embryos

For the germination stage, embryos after desiccation for 0, 1, 4, 7 and 14 days were transferred to germination medium. Based on our previous studies (unpublished data), the optimal germination medium contained ~30 mL of 1/4 LM with 0.6% gellan gum, 2% sucrose, 0.1% activated charcoal, 0.1% casein hydrolysate and 3.42 mm filter‐sterilized L‐glutamine. Thirty embryos were cultured in one Petri dish. Five independent biological replicates were used. In the first week, embryos were maintained in germination medium under a 16‐h photoperiod at a low light intensity (~15 μmol/m^2^/s (LED fluorescent tubes)). Then, the light intensity was increased to 50 μmol/m^2^/s while the photoperiod was unchanged. The culture temperature was kept at 24 ± 1 °C during germination. Germinated embryos were counted after 3 weeks.

### Protein extraction and digestion

Proteomic sequencing was performed by the Beijing Genome Institute (BGI). Total proteins were extracted according to the method of Qiao *et al*. (2012) with some modifications. Somatic embryos (200 mg) were ground to a fine powder in liquid nitrogen and suspended in 500 μL of lysis buffer (40 mm Tris–HCl, 2 m thiourea, 7 m urea, 4% CHAPS, pH 8.5) containing 1 mm phenylmethylsulfonyl fluoride (PMSF), 2 mm EDTA and 10 mm dithiothreitol (DTT), with supersonic extraction for 15 min. The homogenate was centrifuged at 30 000 ***g*** for 20 min at 4 °C. Then, the supernatant was mixed with a fivefold volume of chilled acetone containing 10% (w/v) trichloroacetic acid (TCA) and incubated at −20 °C overnight. The precipitate was vacuum‐dried and again dissolved in 300 μL of lysis buffer (20 mm Tris–HCl, 2 m thiourea, 7 m urea, 4% Nonidet P‐40, pH 8.5) again. After vortex mixing for 2 min, 10 mm DTT (final concentration) was added to the supernatant, then incubated at 56 °C for 1 h and alkylated with 55 mm iodoacetamide (IAM) at 45 °C in a darkroom for 1 h. The supernatant was mixed well with a fivefold volume of chilled acetone and incubated at −20 °C overnight. After centrifugation at 30 000 ***g*** at 4 °C for 20 min, the supernatant was discarded. The protein pellets were vacuum‐dried and dissolved in 500 μL of 0.5 m triethylammonium bicarbonate (TEAB) and centrifuged at 30 000 ***g*** for 15 min at 4 °C. The protein content was assayed using the Bradford method (Bradford, 1976).

### iTRAQ labelling and strong cation exchange (SCX) fractionation

Protein samples containing 100 μg of protein were digested using Trypsin Gold (Promega, Madison, WI) at a protein: trypsin ratio of 20 : 1 at 37 °C for 16 h to obtain peptides. After digestion, the peptides were dried by vacuum centrifugation and resuspended in 0.5 m TEAB. For the sample labelling, 8‐plex iTRAQ reagents (Applied Biosystems, Foster City, CA) were used according to the manufacturer's protocol (Zieske, 2006). For each treatment, there were two biological replicates. Samples were labelled with the iTRAQ tags as follows: Sample D1 (113 and 114 tag), Sample D7 (115 and 116 tag), Sample D14 (117 and 118 tag) and Sample G1 (119 and 121 tag).

Strong cation exchange chromatography was performed on an LC‐20AB HPLC Pump system (Shimadzu, Kyoto, Japan). The iTRAQ‐labelled peptide mixtures were reconstituted in 4 mL buffer A (25% v/v acetonitrile, 25 mm NaH_2_PO_4_, pH 2.7) and loaded onto a 4.6 × 250 mm Ultremex SCX column containing 5‐μm particles (Phenomenex, Torrance, CA). The peptides were eluted at 1 mL/min with elution buffer B (25% v/v acetonitrile, 25 mm NaH_2_PO_4_, 1 m KCl, pH 2.7). Elution was monitored by measuring the absorbance at 214 nm, and fractions were collected every 1 min. The eluted peptides were pooled into 20 fractions, desalted on a Strata X C18 column (Phenomenex) and vacuum‐dried before LC‐ESI‐MS/MS analysis.

### LC‐ESI‐MS/MS analysis

Each fraction was resolved in solvent A (5% acetonitrile, 0.1% formic acid) and centrifuged at 20 000 ***g*** for 10 min, and the average final concentration of peptide was ~0.5 μg/μL. The supernatant was separated using an LC‐20AD Nano‐HPLC (Shimadzu) with an autosampler onto a 2‐cm C18 trap column. Then, the peptides were eluted onto a 10‐cm analytical C18 column (inner diameter 75 μm) packed in‐house. The samples were loaded at 8 μL/min for 4 min, and then the 35‐min linear gradient was run at 300 nL/min starting from 2% to 35% solvent B (95% acetonitrile, 0.1% formic acid), followed by ramping up to 60% solvent B over 5 min, up to 80% in 2 min and maintained for 4 min, then finally restored to 5% in 1 min.

Data were acquired on a TripleTOF 5600 System (AB SCIEX, Concord, Ontario, Canada), using an ion spray voltage of 2.5 kV, nitrogen gas at 30 psi, nebulizer gas at 15 psi and an interface heater temperature of 150 °C. The MS was operated in high‐resolution mode (>30 000 FWHM) for TOF MS scans. For information dependent data acquisition (IDA), survey scans were acquired in 250 ms, and as many as 30 product ion scans were collected if they exceeded a threshold of 120 counts/s and had a 2 +  to 5 +  charge state. The total cycle time was fixed at 3.3 s, and the Q2 transmission window was 100 Da for 100%. Four time bins were summed for each scan at a pulse frequency of 11 kHz, by monitoring of 40‐GHz multichannel TDC detector using four‐anode channel detection. A sweeping collision energy setting of 35 ± 5 eV, coupled with the iTRAQ adjust rolling collision energy, was applied to all precursor ions for collision‐induced dissociation. Dynamic exclusion was set at 1/2 of the peak width (15 s), and then the precursor was refreshed off the exclusion list.

### iTRAQ protein identification and quantification

Raw data files were converted into MGF files using Proteome Discoverer 1.2 (PD 1.2, Thermo, 5600 msconverter) and the files were searched. Proteins were identified using the Mascot search engine (Matrix Science, London, UK; version 2.3.02) against the TreeGenes nonredundant sequence database (http://dendrome.ucdavis.edu/treegenes/protein/prot_summary.php) containing 18 253 sequences. The search parameters were as follows: a mass tolerance of 0.1 Da (ppm) was permitted for intact peptide masses, and 0.05 Da for fragmented ions, with an allowance for one missed cleavage in the trypsin digests; Gln‐>pyro‐Glu (N‐term Q), oxidation (M) and iTRAQ 8‐plex (Y) were the potential variable modifications, and carbamidomethyl (C), iTRAQ 8‐plex (N‐term) and iTRAQ 8‐plex (K) were the fixed modifications. The charge states of the peptides were set to +2 and +3. Specifically, an automatic decoy database search was performed in Mascot by choosing the decoy checkbox in which a random sequence of the database was generated and tested for raw spectra as well as the real database. To reduce the probability of false peptide identification, only peptides with significance scores ≥20 at a 99% confidence interval by a Mascot probability analysis greater than ‘identity’ were counted as identified. Each confidently identified protein included at least one unique peptide. For protein quantization, we required that a protein contained at least two unique peptides. The quantitative protein ratios were weighted and normalized by the median ratio in Mascot. A 1.5‐fold cut‐off was set to determine quantitative changes of up‐regulated and down‐regulated proteins, with a *P*‐value < 0.05.

### Bioinformatics analysis of proteomic data

Functional analysis of identified proteins was conducted using GO annotation (http://www.geneontology.org/) and they were categorized according to their molecular function, biological process and cellular component. The identified proteins were further assigned to the Clusters of Orthologous Groups of proteins (COG) database (http://www.ncbi.nlm.nih.gov/COG/) and the Kyoto Encyclopedia of Genes and Genomes (KEGG) database (http://www.genome.jp/kegg/pathway.html). The flat files of *Picea abies*,* Picea glauca* and *Pinus taeda* proteins were each downloaded from UniProt as reference data sets. To use all identified proteins as a reference data set, the *P*‐value that applied a hypergeometric distribution with FDR correction was calculated to obtain significant enrichment GO catalogues. The GO terms showing *P *< 0.05 were considered to be enriched.

All the identified proteins were taken as a reference for GO function entry enrichment significance analysis, which was used to determine whether the biological processes and functions of differentially accumulated proteins were significantly associated. Meanwhile, the corresponding relations between these proteins with KEGG Orthology (KO) IDs were selected from the protein annotation file. Then, these proteins were mapped to KEGG pathways by invoking the KEGG API according to the KO IDs. The KEGG terms showing *P *< 0.05 were considered to be enriched.

### Network analysis

The protein–protein interactions (PPIs) of *A. thaliana* were downloaded from the IntAct database (http://www.ebi.ac.uk/intact/). Then, the protein sequences in these interaction pairs were also obtained from UniProt (www.uniprot.org). All identified proteins of *P. asperata* somatic embryos were mapped to the downloaded proteins of *A. thaliana* using the single‐directional best hit method with an *e*‐value of 1 × 10^−10^ and >30% similarity. By mapping the relations of PPIs in *A. thaliana*, the PPIs of the identified proteins in *P. asperata* somatic embryos were predicted. Furthermore, a predicted network of the identified proteins was constructed with Cytoscape (version: 2.8.3).

### Determination of water, ABA, IAA and H_2_O_2_ content

The water content of somatic embryos was determined gravimetrically before and after partial desiccation. FW defined the weight before PDT, and DW0 defined the fresh weight during PDT. The embryos were dried in an oven at 60 °C for 48 h after desiccation. DW48 defined the dry weight during PDT. The changes of fresh weight during PDT were expressed as DW0/FW (%). The changes of embryonic dry weight during PDT were expressed as DW48/FW (%).

The contents of ABA and IAA were measured as described by Yang *et al*. (2001) and modified as follows. Samples of 100 mg were extracted with 10 mL 80% (v/v) cold methanol containing 1 mm butylated hydroxytoluene as an antioxidant. The extract was incubated at 4 °C overnight. After centrifugation at 10 000 ***g*** for 20 min at 4 °C, the supernatant was passed through Chromosep C_18_ columns (C_18_ Sep‐Park Cartridge; Waters Corp., Millford, MA), prewashed with 10 mL 100% (v/v) methanol, 5 mL 100% (v/v) ether and 5 mL 100% methanol, respectively. The hormone fractions were dried under N_2_ and dissolved in 2 mL 0.01 m phosphate‐buffered saline (PBS) containing 0.1% (v/v) Tween‐20, 0.1% (w/v) gelatin (pH 7.4) for analysis by enzyme‐linked immunosorbent assay (ELISA). The absorbance was recorded at 490 nm. The ABA and IAA contents were expressed as ng/g FW. H_2_O_2_ was assayed as described by Patterson *et al*. (1984) and was expressed as μg/g FW. Catalase activity was determined using the method described by Cui *et al*. (1999).

All data were analysed for significance using analysis of variance (ANOVA), and the differences were compared using Duncan's multiple range test. Percentage data were transformed by arcsine prior to analysis.

## Supporting information


**Figure S1** Distribution of lengths and numbers of peptides, masses, and sequence coverage of proteins.
**Figure S2** Repeatability of proteome biological replicates during PDT using iTRAQ.
**Figure S3** GO cellular component of *P. asperata* somatic embryo proteins.
**Figure S4** GO molecular function of *P. asperata* somatic embryo proteins.
**Figure S5** GO biological process of *P. asperata* somatic embryo proteins.
**Figure S6** Venn diagram of differentially‐accumulated proteins in *P. asperata* somatic embryos during partial desiccation.
**Figure S7** Catalase activity in *P. asperata* somatic embryos on D0, D7, and D14.
**Figure S8** Differentially‐accumulated proteins in the photosynthesis pathway.
**Figure S9** The protein‐protein interaction network between D7 and D0.
**Figure S10** The protein‐protein interaction network between D14 and D7.
**Figure S11** The protein‐protein interaction network between D14 and D0.
**Figure S12** The protein‐protein interaction network between G1 and D7.
**Table S1** Differentially‐accumulated proteins were associated with water‐deficit tolerance.
**Table S2** Differentially‐accumulated proteins associated with photosynthesis in D14 *vs* D7 and G1 *vs* D7.
**Table S3** Fold changes of stress‐related proteins accumulated in both D7 *vs* D0 and D14 *vs* D0.
