# Supplementary material for: Proteomic analysis of stress‐related proteins and metabolic pathways in Picea asperata somatic embryos during partial desiccation
Source: Plant Biotechnol J. 2016 Jul 14;15(1):27–38. doi: 10.1111/pbi.12588 (PMC5253475; doi:10.1111/pbi.12588)
Supplement: Supplementary file 1 — Figure S1 Distribution of lengths and numbers of peptides, masses, and sequence coverage of proteins. Figure S2 Repeatability of proteome biological replicates during PDT using iTRAQ. Figure S3 GO cellular component of P. asperata somatic embryo proteins. Figure S4 GO molecular function of P. asperata somatic embryo proteins. Figure S5 GO biological process of P. asperata somatic embryo proteins. Figure S6 Venn diagram of differentially‐accumulated proteins in P. asperata somatic embryos during partial desiccation. Figure S7 Catalase activity in P. asperata somatic embryos on D0, D7, and D14. Figure S8 Differentially‐accumulated proteins in the photosynthesis pathway. Figure S9 The protein‐protein interaction network between D7 and D0. Figure S10 The protein‐protein interaction network between D14 and D7. Figure S11 The protein‐protein interaction network between D14 and D0. Figure S12 The protein‐protein interaction network between G1 and D7. Table S1 Differentially‐accumulated proteins were associated with water‐deficit tolerance. Table S2 Differentially‐accumulated proteins associated with photosynthesis in D14 vs D7 and G1 vs D7. Table S3 Fold changes of stress‐related proteins accumulated in both D7 vs D0 and D14 vs D0. [file PBI-15-27-s001.doc]

Supporting information


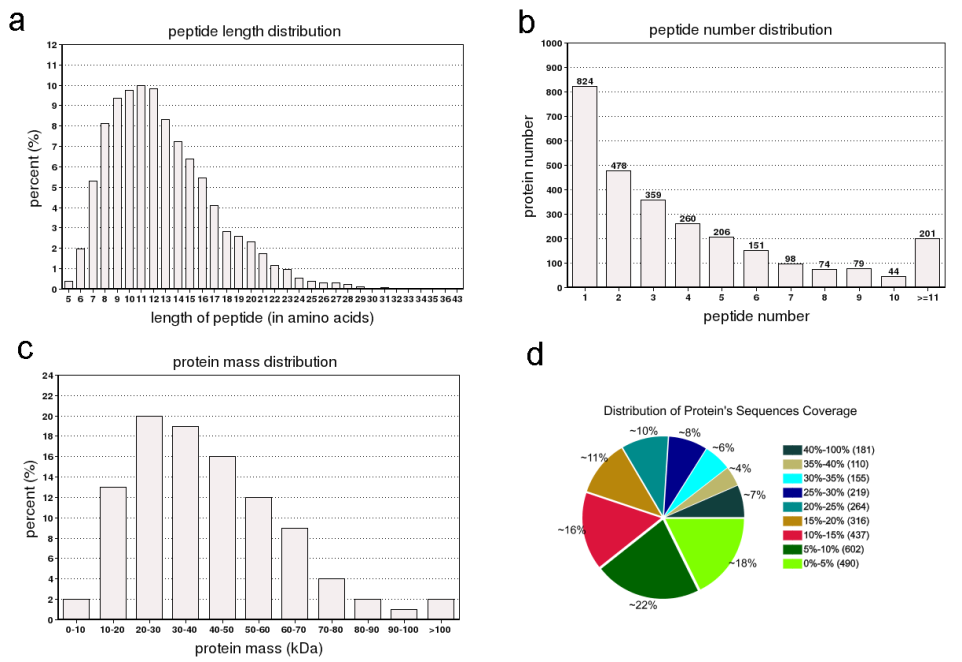


Figure S1 Distribution of lengths and numbers of peptides, masses, and sequence coverage of proteins. (a) the distribution of peptide lengths; (b) the distribution of peptide numbers; (c) the distribution of protein masses; (d) the distribution of protein sequence coverage.


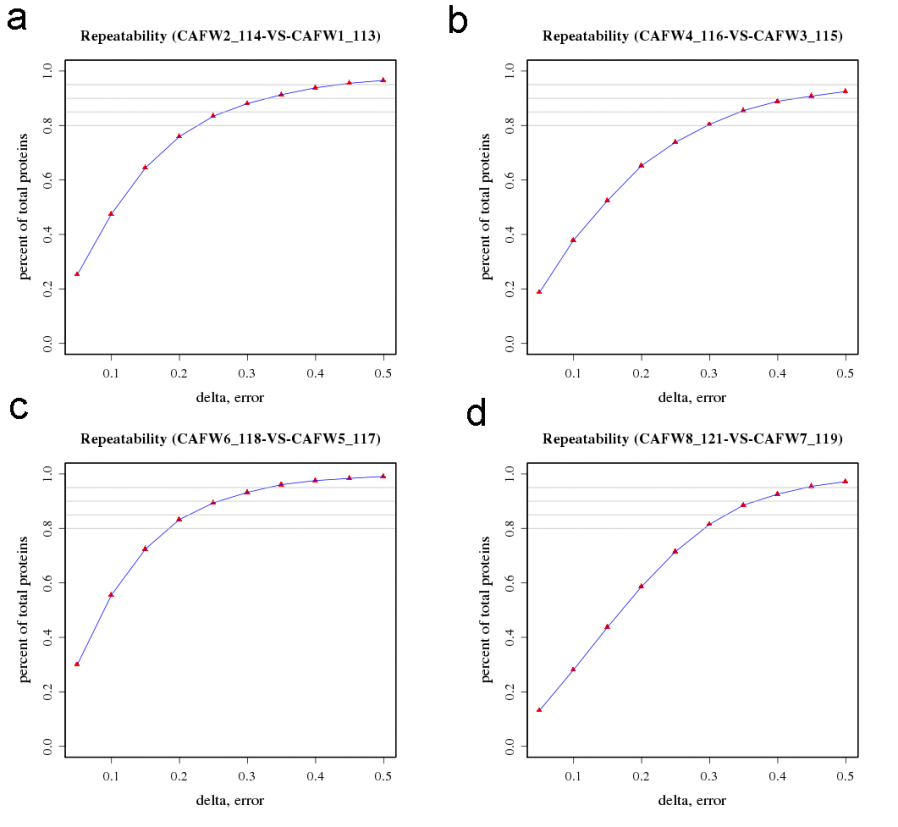


Figure S2 Repeatability of proteome biological replicates during PDT using iTRAQ. (a) the repeatability of *P. asperata* somatic embryos on D0; (b) the repeatability of *P. asperata* somatic embryos on D7; (c) the repeatability of *P. asperata* somatic embryos on D14; (d) the repeatability of *P. asperata* somatic embryos on G1.


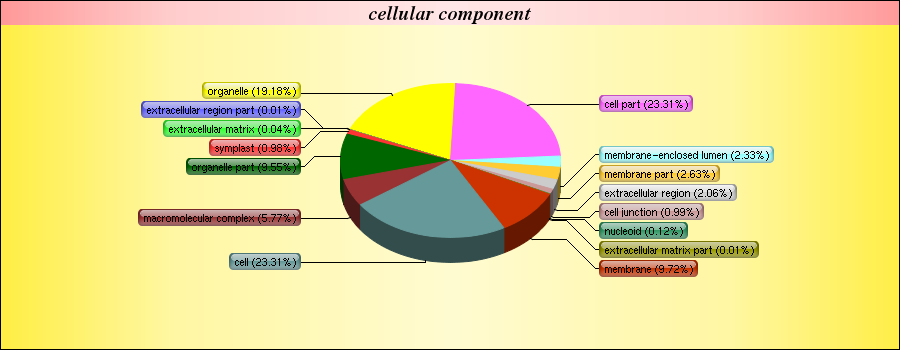


Figure S3 GO cellular component of *P. asperata* somatic embryo proteins


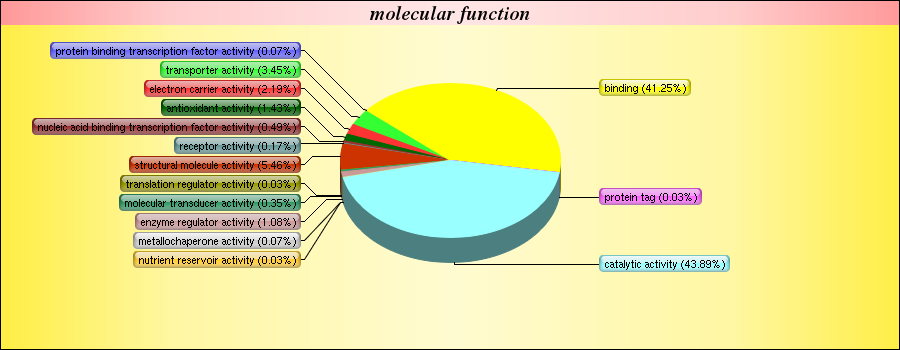


Figure S4 GO molecular function of *P. asperata* somatic embryo proteins.


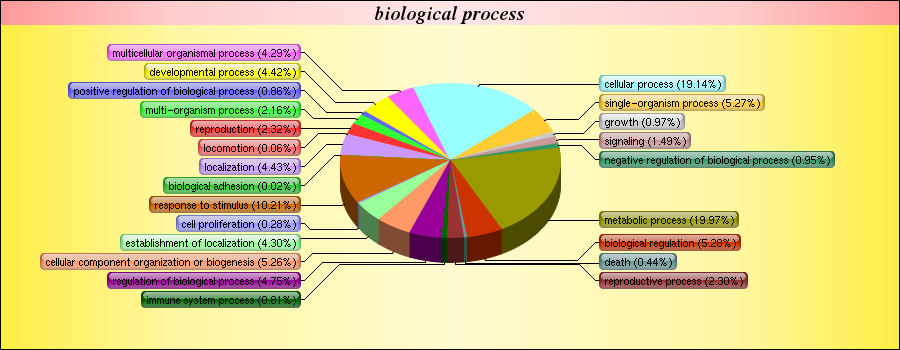


Figure S5 GO biological process of *P. asperata* somatic embryo proteins.


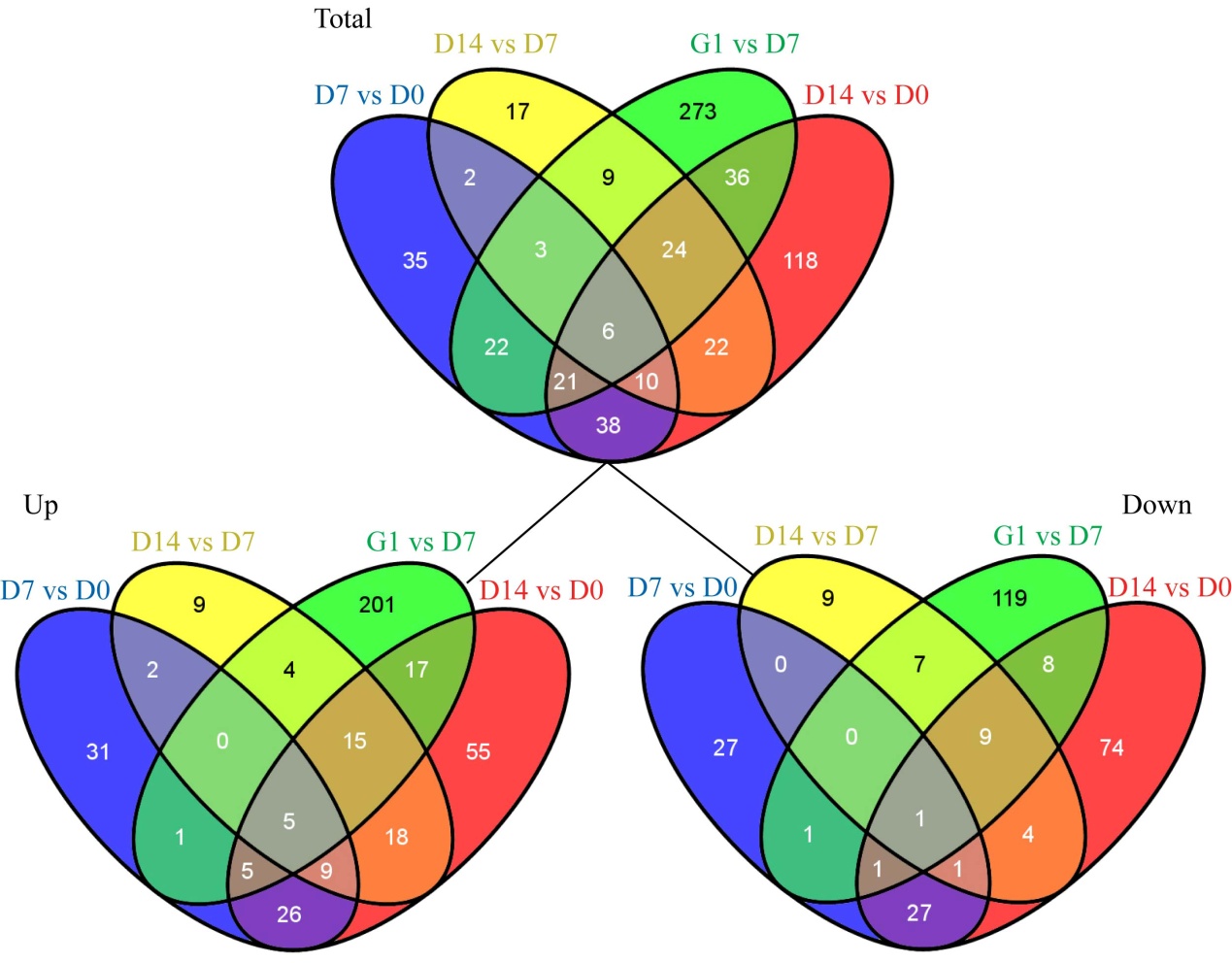


Figure S6 Venn diagram of differentially-accumulated proteins in *P. asperata* somatic embryos during PDT. The Venn diagram showed the number of differentially-accumulated proteins. Total represented the overall differentially-accumulated proteins. Up and Down represented the up-regulated and down-regulated differentially-accumulated proteins, respectively.


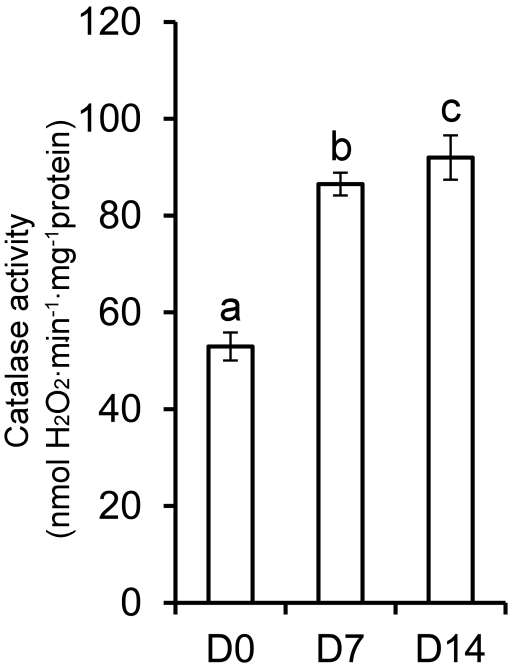


Figure S7 Catalase activity in *P. asperata* somatic embryos on D0, D7, and D14.


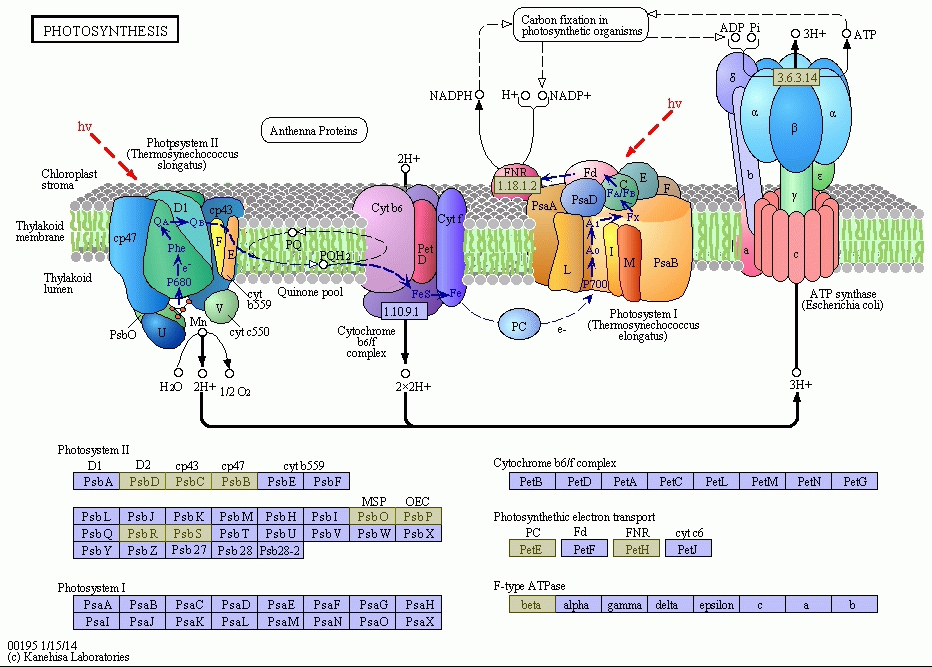


Figure S8 Differentially-accumulated proteins in the photosynthesis pathway. Gray part indicated that the differentially-accumulated proteins were identified.


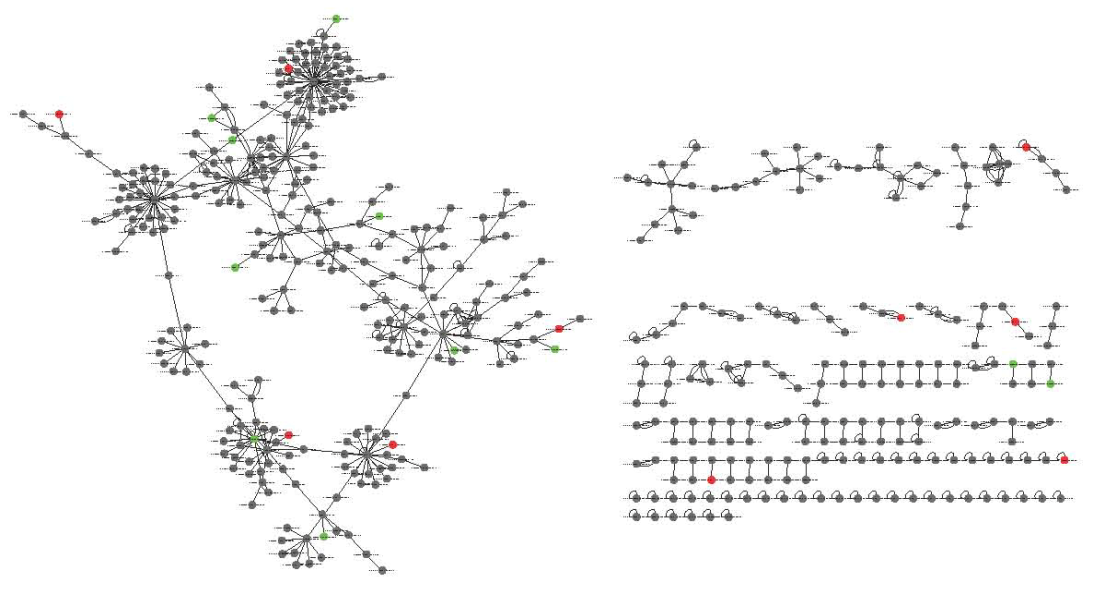


Figure S9 The protein-protein interaction network between D7 and D0.


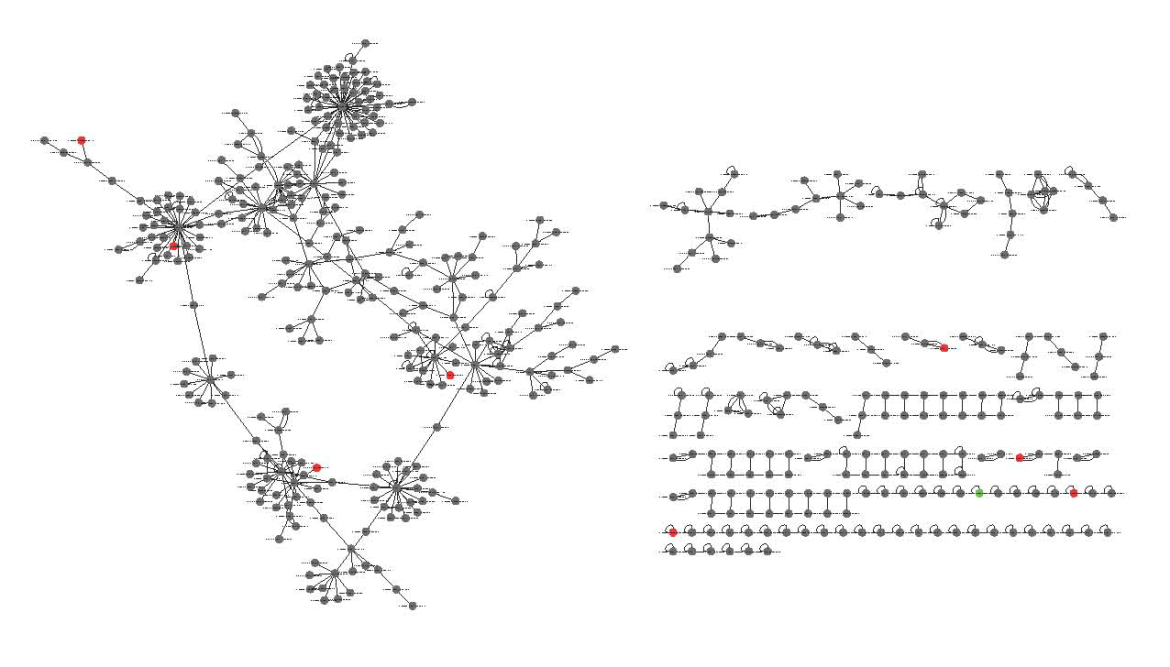


Figure S10 The protein-protein interaction network between D14 and D7.


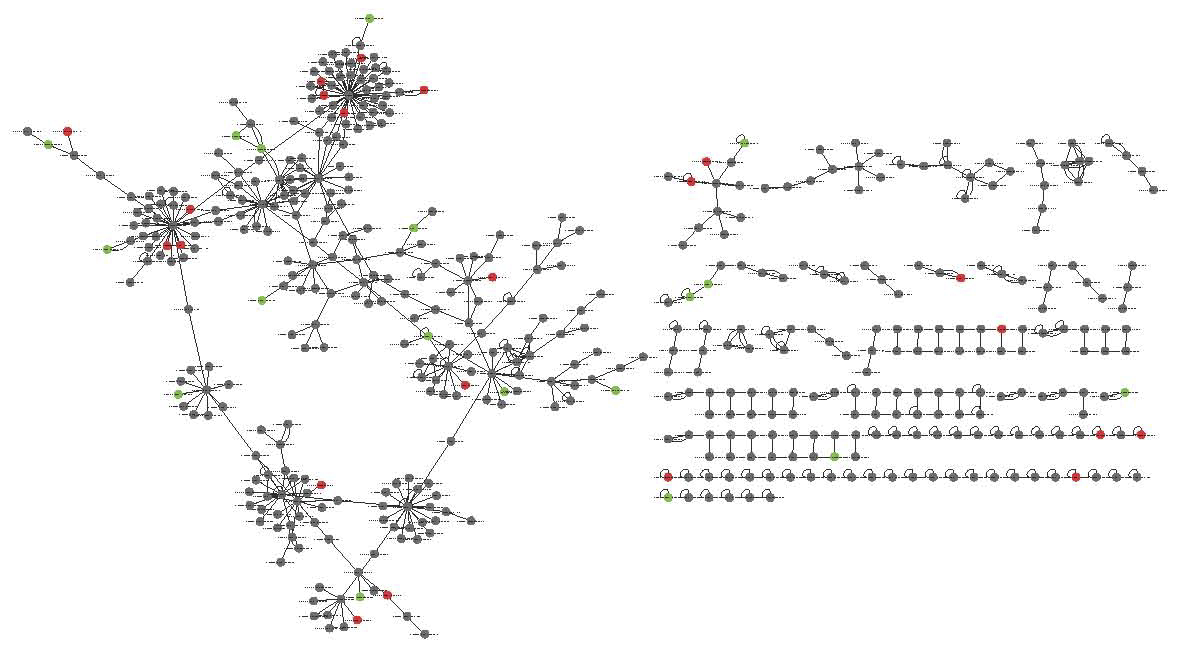


Figure S11 The protein-protein interaction network between D14 and D0.


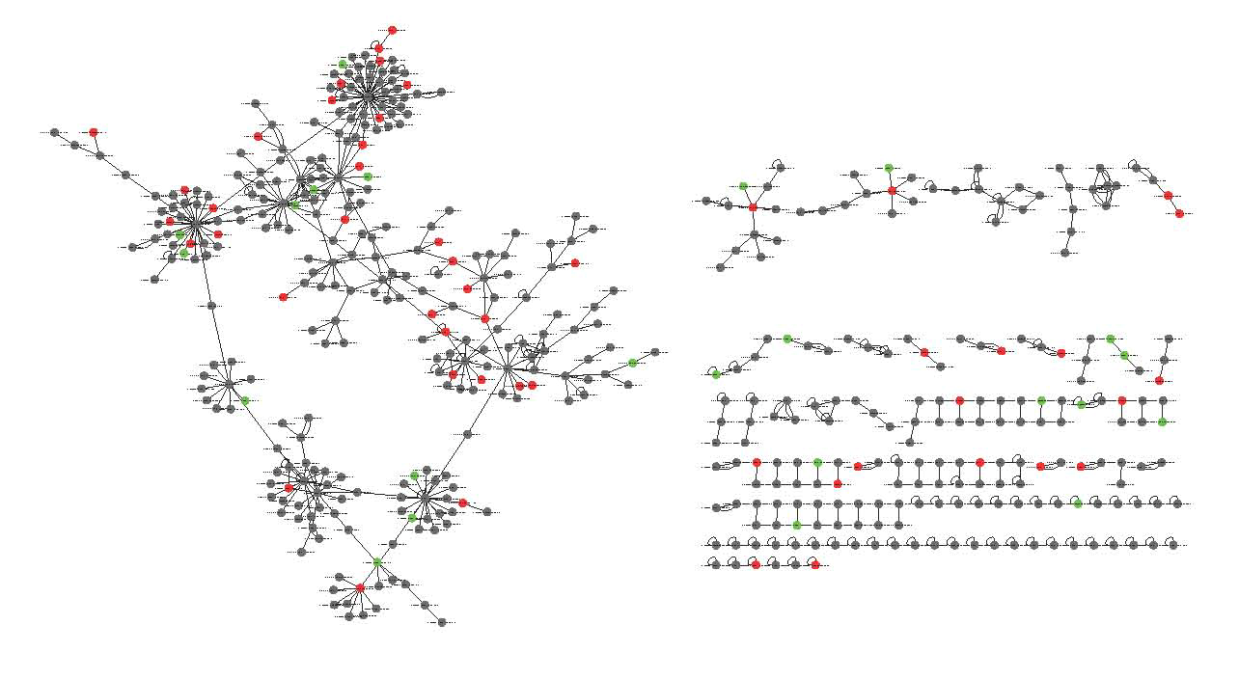


Figure S12 The protein-protein interaction network between G1 and D7.

Table S1 Differentially-accumulated proteins were associated with water deficit tolerance.

| Physiological function | Protein name (accession No.) |  |
| --- | --- | --- |
| Endogenous hormones-related proteins | Auxin-repressed 12.5 kDa protein (Pasi_116782673); | Allene oxide synthase (Pasi_116789220); |
|  | Abscisic stress-ripening protein 2 (Pasi_116779884); |  |
| Osmosis | Aquaporin TIP2-1(Pasi_116779872); | Aquaporin TIP1-1 (Pasi_116782206); |
|  | Probable aquaporin PIP-type pTOM75 (Paab_2258135) |  |
| Defense-related proteins | Pathogenesis-related protein 1 Pasi_116779560; | Wound-induced protein Pasi_116783837; |
|  | Basic endochitinase Paab_33359616; | Chitinase 4 Paab_33359614 |
|  | Chitinase 5 Pasi_148909720 | Basic endochitinase CHB4 Psme_261889446 |
|  | Basic endochitinase C Pasi_116786734 | Osmotin (Pasi_148908401) |
| Antioxidative proteins | Catalase Pisy_170181038 | Catalase isozyme 2 Pasi_116787297 |
|  | Peroxidase 51 Pasi_116780959 | Peroxidase 12 Paab_55057255 |
|  | Glucan endo-1,3-alpha-glucosidase agn1 Pasi_224285168 | Glutathione S-transferase Pasi_116792087 |
|  | Glucan endo-1,3-beta-glucosidase Pasi_116791468 | Alcohol dehydrogenase-like 6 Pasi_148908806 |
|  | Aldehyde dehydrogenase family 7 Pasi_148910753 | Glutathione S-transferase U17 Pasi_116794318 |
|  | Probable glucan 1,3-beta-glucosidase A Pasi_148906180 | L-ascorbate peroxidase 2 Pasi_116793261 |
|  | Isoflavone reductase homolog A622 Pita_1353639 | Glutamate decarboxylase Pasi_224286609 |
|  | Putative oxygen-evolving enhancer protein 2-2 Pasi_116785789 | L-ascorbate oxidase homolog Pasi_116789636 |
|  | Putative UDP-glucuronosyltransferase ugt-46 Pasi_116788605 | Cysteine proteinase Pasi_224285024 |
|  | Probable ornithine aminotransferase Pisy_91680593 | Malate synthase Pasi_116788383 |
|  | Probable trans-2-enoyl-CoA reductase 1 Pasi_116792451 | Strictosidine synthase Pasi_294461975 |
|  | Quinone oxidoreductase-like protein At1g23740 Pasi_224284411 | Cysteine proteinase 3 Pasi_224286306 |
|  | Acyl-coenzyme A oxidase 3 Pasi_224286804 | Chalcone synthase Pita_383154742 |
|  | Serine-glyoxylate aminotransferase Pasi_224284122 | Beta-glucosidase 1 Pasi_148909279 |
|  | Glucose-6-phosphate 1-dehydrogenase Pasi_224286597 | Fumarate hydratase 2 Pasi_116788172 |
|  | Phenylethylamine oxidase Pasi_148906188 | Kynurenine 3-monooxygenase Pasi_224285334 |
|  | Chalcone-flavonone isomerase Pasi_116784315 | Thioredoxin H-type Pasi_116788625 |
|  | Probable mannitol dehydrogenase Pasi_116791508 |  |
| Carbohydrate metabolism | Phosphoenolpyruvate carboxykinase Pasi_294464659 | Aldose 1-epimerase Pasi_116781056 |
|  | NAD-dependent malic enzyme 62 kDa isoform Pasi_148907636 | Sucrose synthase 2 Pasi_306016259 |
|  | Xyloglucan endotransglucosylase/hydrolase protein A Pasi_116791175 |  |
| Molecular chaperones | Small heat shock protein Pasi_224286188 | Class I heat shock protein Pasi_116786112 |
|  | Splicing factor U2af small subunit A Pasi_148908155 | Heat shock 70 kDa protein 10 Pita_383136233 |
|  | 18.1 kDa class I heat shock protein Pita_361068183 |  |
| pyrimidine metabolism | Deoxyuridine 5'-triphosphate nucleotidohydrolase Pasi_116794416 |  |
| Embryogenesis-specific protein | Provicilin Pasi_116788386 | Stem-specific protein Pasi_116782212 |
|  | Tubulin beta-3 chain Pita_383156803 | Tubulin beta-1 chain Pasi_224286571 |
|  | Tubulin alpha chain Pasi_224286798 | 12S seed storage protein Pasi_294464559 |

Table S2 Differentially-accumulated proteins associated with photosynthesis in D14 *vs* D7 and G1*vs* D7.

| Protein name (accession No.) |  |
| --- | --- |
| Chlorophyll a-b binding protein of LHCII type 1 (Paab_607150) ^a^ |  |
| Ribulose bisphosphate carboxylase small chain, chloroplastic (Pasi_116780862) ^ab^ | Quinone oxidoreductase-like protein At1g23740, chloroplastic(Pasi_116786555) ^b^ |
| Chlorophyll a-b binding protein type 2 member 2 (Pisy_227138) ^ab^ | Small heat shock protein, chloroplastic (Pasi_224285764) ^b^ |
| Photosystem II CP43 chlorophyll apoprotein (Pita_228016348) ^a^ | Phospho-2-dehydro-3-deoxyheptonate aldolase 1, chloroplastic (Pasi_116786853) ^b^ |
| Photosystem II CP47 chlorophyll apoprotein (Paab_502524869) ^ab^ | 31 kDa ribonucleoprotein, chloroplastic (Pasi_116787605) ^b^ |
| Oxygen-evolving enhancer protein 1, chloroplastic (Pasi_224284112) ^ab^ | Small heat shock protein, chloroplastic (Pagl_21068482) ^b^ |
| Ferredoxin--NADP reductase, leaf isozyme, chloroplastic (Pasi_116780412) ^a^ | Putative quinone-oxidoreductase homolog, chloroplastic (Pasi_294463889) ^b^ |
| Protochlorophyllide reductase (Pasi_224285652) ^ab^ | Malate dehydrogenase, chloroplastic (Pasi_116786984) ^b^ |
| Sedoheptulose-1,7-bisphosphatase, chloroplastic (Pasi_116786455) ^ab^ | Dihydrodipicolinate reductase 2, chloroplastic (Pasi_148907184) ^b^ |
| Photosystem II 22 kDa protein, chloroplastic (Pasi_116790850) ^a^ | Histidinol dehydrogenase, chloroplastic (Pasi_116787376) ^b^ |
| Chlorophyll a-b binding protein CP26, chloroplastic (Pasi_116783955) ^a^ | Thioredoxin M-type, chloroplastic (Pasi_116784110) ^b^ |
| Photosystem II 10 kDa polypeptide, chloroplastic (Pasi_224285151) ^a^ | Uncharacterized protein At2g37660, chloroplastic (Pasi_116782558) ^b^ |
| Glyceraldehyde-3-phosphate dehydrogenase A, chloroplastic (Pasi_116786948) ^a^ | Clp protease-related protein At4g12060, chloroplastic (Pasi_224285995) ^b^ |
| Photosystem II D2 (Pisy_356997659) ^a^ | SufE-like protein, chloroplastic (Pasi_116789886) ^b^ |
| Ribulose bisphosphate carboxylase/oxygenase activase, chloroplastic (Pasi_116787537) ^ab^ | ATP-dependent Clp protease proteolytic subunit 4, chloroplastic (Pasi_116786218) ^a^ |
| Plastocyanin (Pasi_224284714) ^a^ | 30S ribosomal protein 2, chloroplastic (Pasi_116790995) ^ab^ |
| 50S ribosomal protein L14, chloroplastic (Paab_502524880) ^a^ | 50S ribosomal protein L5, chloroplastic (Pasi_224284364) ^ab^ |
| Light-independent protochlorophyllide reductase subunit (Paab_502524896) ^ab^ | Elongation factor TuB, chloroplastic (Pasi_116788862) ^a^ |
| 25.3 kDa heat shock protein, chloroplastic (Pita_383169809) ^a^ | Small heat shock protein, chloroplastic (Pasi_224286188) ^ab^ |
| Rhodanese-like domain-containing protein 4, chloroplastic (Pasi_148906885) ^a^ | 50S ribosomal protein L3, chloroplastic (Pasi_116780925) ^a^ |
| ATP synthase subunit beta, chloroplastic (Paab_3913118) ^a^ | Small heat shock protein, chloroplastic (Pasi_148907259) ^ab^ |
| Protein THYLAKOID FORMATION1, chloroplastic (Pasi_116782546) ^a^ | 29 kDa ribonucleoprotein, chloroplastic (Pasi_116786295) ^ab^ |
| Glyceraldehyde-3-phosphate dehydrogenase A, chloroplastic (Pasi_224286483) ^a^ | Glyceraldehyde-3-phosphate dehydrogenase A, chloroplastic (Pasi_224286603) ^a^ |
| Allene oxide synthase, chloroplastic (Pasi_116789220) ^a^ | 30S ribosomal protein S5, chloroplastic (Pasi_116787539) ^ab^ |
| Light-independent protochlorophyllide reductase subunit B (Paab_502524832) ^a^ | 50S ribosomal protein L2, chloroplastic (Paab_6093986) ^a^ |
| 30S ribosomal protein S1, chloroplastic (Pasi_116790590) ^ab^ | 50S ribosomal protein L9, chloroplastic (Pasi_116789745) ^a^ |
| Glutamate-1-semialdehyde 2,1-aminomutase,chloroplastic (Pita_383145075) ^ab^ |  |

a, represented differentially-accumulated proteins of photosynthesis in D14 *vs* D7; b, represented differentially-accumulated proteins of photosynthesis in G1*vs* D7.

Table S3 Fold-changes of stress-related proteins accumulated in both D7 *vs* D0 and D14 *vs* D0

| Accession | Description | Score ^a^ | Cov(%) ^b^ | Peptide ^c^ | D7 VS D0 | D14 VS D0 |
| --- | --- | --- | --- | --- | --- | --- |
| protein_Pasi_116779560 | Pathogenesis-related protein 1 | 969 | 60.2 | 9 | 4.981 | 4.905 |
| protein_Pagl_76782066 | Pathogenesis-related protein 1 | 462 | 52.6 | 9 | 4.697 | 3.652 |
| protein_Pasi_116783837 | Wound-induced protein | 165 | 11.9 | 1 | 4.1 | 8.202 |
| protein_Pasi_116782673 | Auxin-repressed 12.5 kDa protein | 569 | 56.3 | 5 | 3.212 | 2.855 |
| protein_Pasi_116792087 | Glutathione S-transferase | 921 | 34.9 | 9 | 2.872 | 8.51 |
| protein_Pasi_116779872 | Aquaporin TIP2-1 | 63 | 7.6 | 2 | 2.751 | 4.56 |
| protein_Pasi_148908806 | Alcohol dehydrogenase-like | 2962 | 38.7 | 12 | 2.676 | 3.838 |
| protein_Pasi_148906180 | Probable glucan 1,3-beta-glucosidase | 1018 | 33.1 | 12 | 2.659 | 3.522 |
| protein_Paab_33359614 | Chitinase 4 | 781 | 16.7 | 4 | 2.6 | 5.304 |
| protein_Pasi_116780862 | Ribulose bisphosphate carboxylase small chain, chloroplastic | 153 | 21.2 | 5 | 2.577 | 9.281 |
| protein_Pasi_116782084 | Alcohol dehydrogenase-like | 2538 | 35.3 | 11 | 2.412 | 4.476 |
| protein_Pasi_116786734 | Basic endochitinase C | 509 | 14.8 | 3 | 2.32 | 3.291 |
| protein_Paab_55057255 | Peroxidase 12 | 176 | 13.3 | 4 | 2.276 | 1.669 |
| protein_Pasi_294464659 | Phosphoenolpyruvate carboxykinase [ATP] | 484 | 40.3 | 7 | 2.06 | 2.944 |
| protein_Pasi_224285168 | Glucan endo-1,3-alpha-glucosidase agn1 | 195 | 10.5 | 4 | 2.025 | 3.225 |
| protein_Pasi_116791556 | Isoflavone reductase homolog A622 | 883 | 28.3 | 9 | 1.997 | 5.052 |
| protein_Pasi_116794416 | Deoxyuridine 5'-triphosphate nucleotidohydrolase | 980 | 49.5 | 7 | 1.988 | 2.504 |
| protein_Pasi_116788625 | Thioredoxin H-type | 273 | 35.8 | 4 | 1.984 | 1.66 |
| protein_Pasi_116780925 | 50S ribosomal protein L3, chloroplastic | 213 | 13.9 | 4 | 1.974 | 2.715 |
| protein_Pasi_116794318 | Glutathione S-transferase U17 | 853 | 44.4 | 10 | 1.873 | 1.694 |
| protein_Pasi_224284990 | Alcohol dehydrogenase-like 6 | 600 | 12.9 | 5 | 1.845 | 3.146 |
| protein_Pasi_224284411 | Quinone oxidoreductase-like protein, chloroplastic | 948 | 29.4 | 7 | 1.826 | 2.346 |
| protein_Pasi_116784656 | Probable glutathione S-transferase | 315 | 21 | 5 | 1.739 | 2.509 |
| protein_Pasi_116792041 | Alpha,alpha-trehalose-phosphate synthase | 217 | 14.5 | 2 | 1.738 | 2.009 |
| protein_Pasi_116793276 | Mugineic-acid 3-dioxygenase | 49 | 6.3 | 3 | 1.709 | 1.643 |
| protein_Pasi_224284112 | Oxygen-evolving enhancer protein, chloroplastic | 1760 | 44.6 | 11 | 1.709 | 7.7 |
| protein_Pasi_294461975 | Strictosidine synthase | 440 | 30.3 | 7 | 1.706 | 1.797 |
| protein_Pasi_224286597 | Glucose-6-phosphate 1-dehydrogenase, cytoplasmic isoform 2 | 247 | 22.8 | 10 | 1.693 | 1.65 |
| protein_Pasi_116792168 | Thylakoid lumenal 15 kDa protein, chloroplastic | 178 | 24.5 | 4 | 1.691 | 1.961 |
| protein_Pasi_116788386 | Provicilin | 473 | 19.4 | 5 | 1.672 | 3.962 |
| protein_Pasi_116779884 | Abscisic stress-ripening protein | 415 | 30.5 | 3 | 1.671 | 2.097 |
| protein_Pasi_224285151 | Photosystem II 10 kDa polypeptide, chloroplastic | 233 | 34.7 | 5 | 1.649 | 5.316 |
| protein_Pita_383143100 | Phospholipase D alpha 2 | 37 | 7.5 | 1 | 1.635 | 5.311 |
| protein_Pisy_170181038 | Catalase | 1097 | 26 | 11 | 1.611 | 1.918 |
| protein_Pasi_116785789 | Putative oxygen-evolving enhancer protein 2-2 | 173 | 24.6 | 6 | 1.598 | 6.509 |
| protein_Pasi_224286188 | Small heat shock protein, chloroplastic | 1176 | 24.8 | 5 | 1.572 | 2.161 |
| protein_Pasi_116786790 | Aldehyde dehydrogenase family 3 member H1 | 99 | 8.2 | 3 | 1.56 | 1.635 |
| protein_Pasi_116787297 | Catalase isozyme 2 | 1093 | 27.2 | 12 | 1.555 | 2.06 |
| protein_Pasi_116786112 | Class I heat shock protein | 113 | 28.8 | 5 | 1.533 | 2.138 |
| protein_Pasi_148908401 | Osmotin | 75 | 7.1 | 1 | 1.528 | 2.103 |
| protein_Pasi_116790850 | Photosystem II 22 kDa protein, chloroplastic | 190 | 14.3 | 3 | 1.522 | 4.328 |
| protein_Pisy_91680593 | Probable ornithine aminotransferase | 188 | 10.3 | 5 | 1.517 | 1.685 |
| protein_Pasi_116788605 | Putative UDP-glucuronosyltransferase ugt-46 | 225 | 10.8 | 4 | 1.504 | 1.747 |
| protein_Pasi_224285178 | Nascent polypeptide-associated complex subunit alpha-like protein | 1499 | 33 | 5 | 0.645 | 0.612 |
| protein_Pasi_116791505 | Stem-specific protein | 83 | 12.4 | 3 | 0.638 | 0.625 |
| protein_Pasi_116788244 | Acidic leucine-rich nuclear phosphoprotein 32-related protein | 672 | 21.9 | 7 | 0.637 | 0.625 |
| protein_Pasi_116792297 | Protein SPIRAL1-like 3 | 163 | 16.7 | 1 | 0.626 | 0.573 |
| protein_Pasi_224286734 | DNA-binding protein | 215 | 17.2 | 2 | 0.62 | 0.507 |
| protein_Pasi_224286899 | Histone H1 | 138 | 21.4 | 4 | 0.617 | 0.506 |
| protein_Pasi_116787420 | Histone H1 | 361 | 7.5 | 2 | 0.614 | 0.443 |
| protein_Pasi_224286155 | 26S proteasome non-ATPase regulatory subunit 4 | 425 | 23.5 | 8 | 0.6 | 0.562 |
| protein_Paab_335885160 | Protein MOTHER of FT and TF 1 | 438 | 41.1 | 4 | 0.598 | 0.474 |
| protein_Pita_383146025 | Cinnamoyl-CoA reductase 2 | 171 | 27.9 | 2 | 0.59 | 0.482 |
| protein_Pisy_1237083 | Phytochrome | 104 | 3.1 | 4 | 0.58 | 0.454 |
| protein_Pasi_116782335 | 40S ribosomal protein S12 | 342 | 14.2 | 1 | 0.568 | 0.638 |
| protein_Pasi_148906576 | BURP domain-containing protein 16 | 218 | 4.8 | 2 | 0.559 | 0.447 |
| protein_Pasi_294463648 | Acyl-[acyl-carrier-protein] desaturase, chloroplastic | 311 | 25.6 | 5 | 0.553 | 0.45 |
| protein_Pasi_224286440 | Protein translation factor SUI1 homolog | 265 | 11.5 | 1 | 0.537 | 0.653 |
| protein_Pasi_116787087 | Putative DNA repair protein | 732 | 19.2 | 5 | 0.521 | 0.455 |
| protein_Pita_383173198 | Protein argonaute PNH1 | 109 | 8.3 | 1 | 0.519 | 0.364 |
| protein_Pasi_116787300 | 1-phosphatidylinositol 3-phosphate 5-kinase | 174 | 14.3 | 5 | 0.518 | 0.531 |
| protein_Pasi_116790333 | Acyl-CoA-binding protein | 181 | 52.9 | 5 | 0.5 | 0.574 |
| protein_Pasi_116780772 | Density-regulated protein homolog | 124 | 15.5 | 2 | 0.49 | 0.43 |
| protein_Pasi_294464103 | DNA-directed RNA polymerase II subunit RPB3-A | 191 | 20.9 | 5 | 0.449 | 0.439 |
| protein_Pasi_116793156 | 22.7 kDa class IV heat shock protein | 112 | 37 | 4 | 0.445 | 0.462 |

a, protein score; b, sequence coverage; c, peptide number
